# Four new species of *Cichlidogyrus* (Platyhelminthes, Monopisthocotyla, Dactylogyridae) from Lake Victoria haplochromine cichlid fishes, with the redescription of *C. bifurcatus* and *C. longipenis*[Fn FN1]

**DOI:** 10.1051/parasite/2024039

**Published:** 2024-08-07

**Authors:** Tiziana P. Gobbin, Maarten P.M. Vanhove, Ole Seehausen, Martine E. Maan, Antoine Pariselle

**Affiliations:** 1 Division of Aquatic Ecology & Evolution, Institute of Ecology and Evolution, University of Bern Baltzerstrasse 6 3012 Bern Switzerland; 2 Groningen Institute for Evolutionary Life Sciences, University of Groningen Nijenborgh 7 9747 AG Groningen The Netherlands; 3 Department of Fish Ecology and Evolution, Centre of Ecology, Evolution and Biogeochemistry, Eawag, Swiss Federal Institute of Aquatic Science and Technology Seestrasse 79 6047 Kastanienbaum Switzerland; 4 Research Group Zoology: Biodiversity & Toxicology, Centre for Environmental Sciences, Hasselt University Agoralaan Gebouw D 3590 Diepenbeek Belgium; 5 Laboratory of Biodiversity and Evolutionary Genomics, Department of Biology, KU Leuven Charles Deberiotstraat 32 3000 Leuven Belgium; 6 Zoology Unit, Finnish Museum of Natural History, University of Helsinki P.O. Box 17 00014 Helsinki Finland; 7 ISEM, CNRS, Université de Montpellier, IRD place Eugène Bataillon 34090 Montpellier France; 8 Laboratory of Biodiversity, Ecology and Genome, Faculty of Sciences, Mohammed V University 4 avenue Ibn Battouta B.P. 1014 RP 10000 Rabat Morocco

**Keywords:** African Great Lakes, Biodiversity, Cichlidae, Dactylogyridea, Haplochromini, Parasite

## Abstract

African cichlids are model systems for evolutionary studies and host-parasite interactions, because of their adaptive radiations and because they harbour many species of monogenean parasites with high host-specificity. Five locations were sampled in southern Lake Victoria: gill-infecting monogeneans were surveyed from 18 cichlid species belonging to this radiation superflock and two others representing two older and distantly related lineages. We found one species of Gyrodactylidae, *Gyrodactylus sturmbaueri* Vanhove, Snoeks, Volckaert & Huyse, 2011, and seven species of Dactylogyridae. Four are described herein: *Cichlidogyrus pseudodossoui* n. sp., *Cichlidogyrus nyanza* n. sp., *Cichlidogyrus furu* n. sp., and *Cichlidogyrus vetusmolendarius* n. sp. Another *Cichlidogyrus* species is reported but not formally described (low number of specimens, morphological similarity with *C. furu* n. sp.). Two other species are redescribed: *C. bifurcatus* Paperna, 1960 and *C. longipenis* Paperna & Thurston, 1969. Our results confirm that the monogenean fauna of Victorian littoral cichlids displays lower species richness and lower host-specificity than that of Lake Tanganyika littoral cichlids. In *C. furu* n. sp., hooks V are clearly longer than the others, highlighting the need to re-evaluate the current classification system that considers hook pairs III–VII as rather uniform. Some morphological features of *C. bifurcatus*, *C. longipenis*, and *C. nyanza* n. sp. suggest that these are closely related to congeners that infect other haplochromines. Morphological traits indicate that representatives of *Cichlidogyrus* colonised Lake Victoria haplochromines or their ancestors at least twice, which is in line with the Lake Victoria superflock being colonised by two cichlid tribes (Haplochromini and Oreochromini).

## Abstract (fr)

Les cichlidés africains sont des systèmes modèles pour les études évolutives et les interactions hôtes-parasites, en raison de leurs radiations adaptatives et parce qu’ils hébergent de nombreuses espèces de monogènes parasites avec une spécificité d’hôte étroite. Cinq sites ont été échantillonnés dans le sud du lac Victoria, les monogènes infectant les branchies ont été étudiés chez 18 espèces de cichlidés appartenant à ce superflock et de deux autres espèces représentant deux lignées plus anciennes et éloignées. Nous avons trouvé une espèce de Gyrodactylidae, *Gyrodactylus sturmbaueri* Vanhove, Snoeks, Volckaert & Huyse, 2011, et sept espèces de Dactylogyridae. Quatre sont décrites ici : *Cichlidogyrus pseudodossoui* n. sp., *Cichlidogyrus nyanza* n. sp., *Cichlidogyrus furu* n. sp. et *Cichlidogyrus vetusmolendarius* n. sp. Une autre espèce de *Cichlidogyrus* est signalée mais non formellement décrite (faible nombre de spécimens, similarité morphologique avec *C. furu* n. sp.). Deux autres espèces sont redécrites : *C. bifurcatus* Paperna, 1960 et *C. longipenis* Paperna & Thurston, 1969. Nos résultats confirment que la faune des monogènes des cichlidés du littoral du lac Victoria présente une richesse en espèces et une spécificité d’hôte inférieures à celles des cichlidés du littoral du lac Tanganyika. Chez *C. furu* n. sp., les crochets V sont clairement plus longs que les autres, ce qui souligne la nécessité de réévaluer le système de classification actuel qui considère que les crochets III-VII sont plutôt uniformes. Certaines caractéristiques morphologiques de *C. bifurcatus*, *C. longipenis* et *C. nyanza* n. sp. suggèrent que ceux-ci sont étroitement liés aux congénères qui infectent les autres haplochrominés. Les traits morphologiques indiquent que les représentants de *Cichlidogyrus* ont colonisé les haplochrominés du lac Victoria ou leurs ancêtres au moins deux fois, ce qui concorde avec le fait que le superflock du lac Victoria a été colonisé par deux tribus de cichlidés (Haplochromini et Oreochromini).

## Introduction

Cichlid fish (Cichlidae) form one of the most species-rich families of vertebrates, occurring mainly in rivers and lakes in Africa and South America. They underwent spectacular adaptive radiations in many African lakes, including lakes Tanganyika, Malawi, and Victoria [10, 23, 53]. The species flocks that evolved in the African Great Lakes display a large diversity in morphology, ecology and behaviour, and high levels of endemism [10, 23, 50, 56, 62, 63]. There are also many cases in which cichlids failed to radiate upon colonising lakes [53, 62, 63]. Together, these characteristics have made African cichlids a rewarding model system for studying adaptation and speciation [23, 24]. In recent years, evidence has accumulated that the diversity of cichlids in the African Great Lakes is also associated with diversity of their parasites [15, 22, 44, 59]. Monogenean flatworms are a promising parasite group to study whether and how the diversification processes in cichlids and their parasites influence each other [41, 58]. This is because of their species richness in African cichlids, their narrow host-specificity compared to other cichlid parasites, and their direct lifecycle.

Most studies of monogenean parasites of African Great Lake cichlids have focused on Lake Tanganyika, the oldest of the three Great Lakes, that also has by far the oldest cichlid radiations (e.g. [42, 59]). From this lake, 39 species of *Cichlidogyrus* Paperna, 1960 [35, 46] and three species of *Gyrodactylus* von Nordmann, 1832 [60] have been described from cichlids. Of these, only one species from each genus has been found also on non-Tanganyikan hosts: *Cichlidogyrus mbirizei* Muterezi Bukinga, Vanhove, Van Steenberge & Pariselle, 2012 and *Gyrodactylus sturmbaueri* Vanhove, Snoeks, Volckaert & Huyse, 2011. These two species were reported for the first time outside of Lake Tanganyika by Lerssutthichawal et al*.* [25] in a cultured *Oreochromis* hybrid in Thailand, and by Zahradníčková et al*.* [66] in *Pseudocrenilabrus philander* (Weber, 1897) in Zimbabwe and South Africa, respectively. Conversely, only two monogenean species previously known from other cichlids outside Lake Tanganyika have been observed in a cichlid endemic to the Tanganyika basin: *Cichlidogyrus halli* (Price & Kirk, 1967) and *Scutogyrus longicornis* (Paperna & Thurston, 1969), both on *Oreochromis tanganicae* (Günther, 1984). This indicates that the monogenean assemblage of Tanganyika cichlids is quite distinct from that of other cichlids. Here, we focus on Lake Victoria, where the monogenean fauna has been investigated less extensively. Research on its monogeneans already peaked in the 1960s–70s [37, 42], yielding ten species of *Cichlidogyrus*, two species of *Gyrodactylus*, and a single species of *Scutogyrus* Pariselle and Euzet, 1985, all of which are also found on cichlids outside the Lake Victoria region, with the exception of *Cichlidogyrus longipenis* Paperna & Thurston, 1969. Only recently, Lake Victoria’s monogeneans received attention again, namely in ecological parasitology [15–17, 22, 26, 27]. Two of these studies distinguished *Cichlidogyrus* at the species level [15–17] and suggest that the species richness, level of endemism, and host-specificity of cichlid-infecting monogeneans may be lower in Lake Victoria than in Lake Tanganyika, a feature that Pariselle et al*.* [42] suggested to be linked to the younger age of the Lake Victoria cichlid species flock.

Lake Victoria is the youngest, and also the shallowest and most turbid of the three Great Lakes. It was completely dry until about 14,600 years ago [20]. Most of its current cichlid fauna evolved *in situ* after that dry period [19, 30, 55]. The Lake Victoria cichlid superflock evolved from a hybrid swarm derived from at least two riverine lineages that colonised the lake [30, 54]. This provided the genetic variation that, together with ample ecological opportunities, allowed rapid speciation [28, 49, 52]. Current cichlid species display a wide range of trophic specialisations [2, 3, 18, 29, 48, 51, 65]. Older cichlid lineages (only distantly related to the species that radiated in the lake) also colonised the lake, but did not speciate: *Astatoreochromis alluaudi* Pellegrin, 1904, *Pseudocrenilabrus multicolor* (Schöller, 1903), *Oreochromis variabilis* (Boulenger, 1906), and *Oreochromis esculentus* (Graham, 1928). These are currently sympatric with species of the Lake Victoria superflock.

Pending a global revision of the species-rich genus *Cichlidogyrus* using genetic data (more than 130 valid species described today: [12, 40, 45, 46]), the species belonging to this genus are grouped together by morphological similarities to facilitate their systematic analysis [43, 61]. Closely related species within the genus can be grouped by the morphology of their reproductive apparatus, whereas genera and subgeneric categories are more easily grouped by the morphology of their haptoral hard parts, one of the first criteria being the relative length of the hooks (i.e., total length of hooks I, III–VII relative to total length of hooks II [39, 40]). In the present study, for the first time in over 40 years, we systematically survey the monogenean fauna infecting the three anciently divergent haplochromine cichlid lineages of Lake Victoria: the radiation lineage (represented here by 18 species) and the two lineages that did not radiate (*Astatoreochromis alluaudi* and *Pseudocrenilabrus multicolor*). This results in four formal taxonomic descriptions and two redescriptions.

## Materials and methods

**Figure 1:**
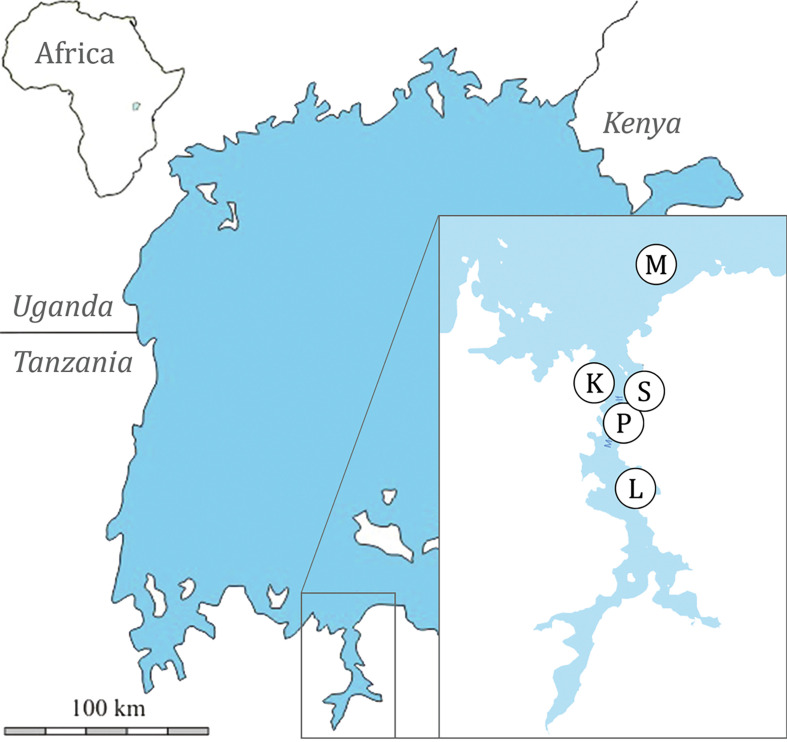
Geographical location of the five sampling sites in southern Lake Victoria, Tanzania: rocky islands Makobe (M), Kissenda (K), Python (P), Luanso (L), and the Sweya swampy inlet stream (S).

**Figure 2:**
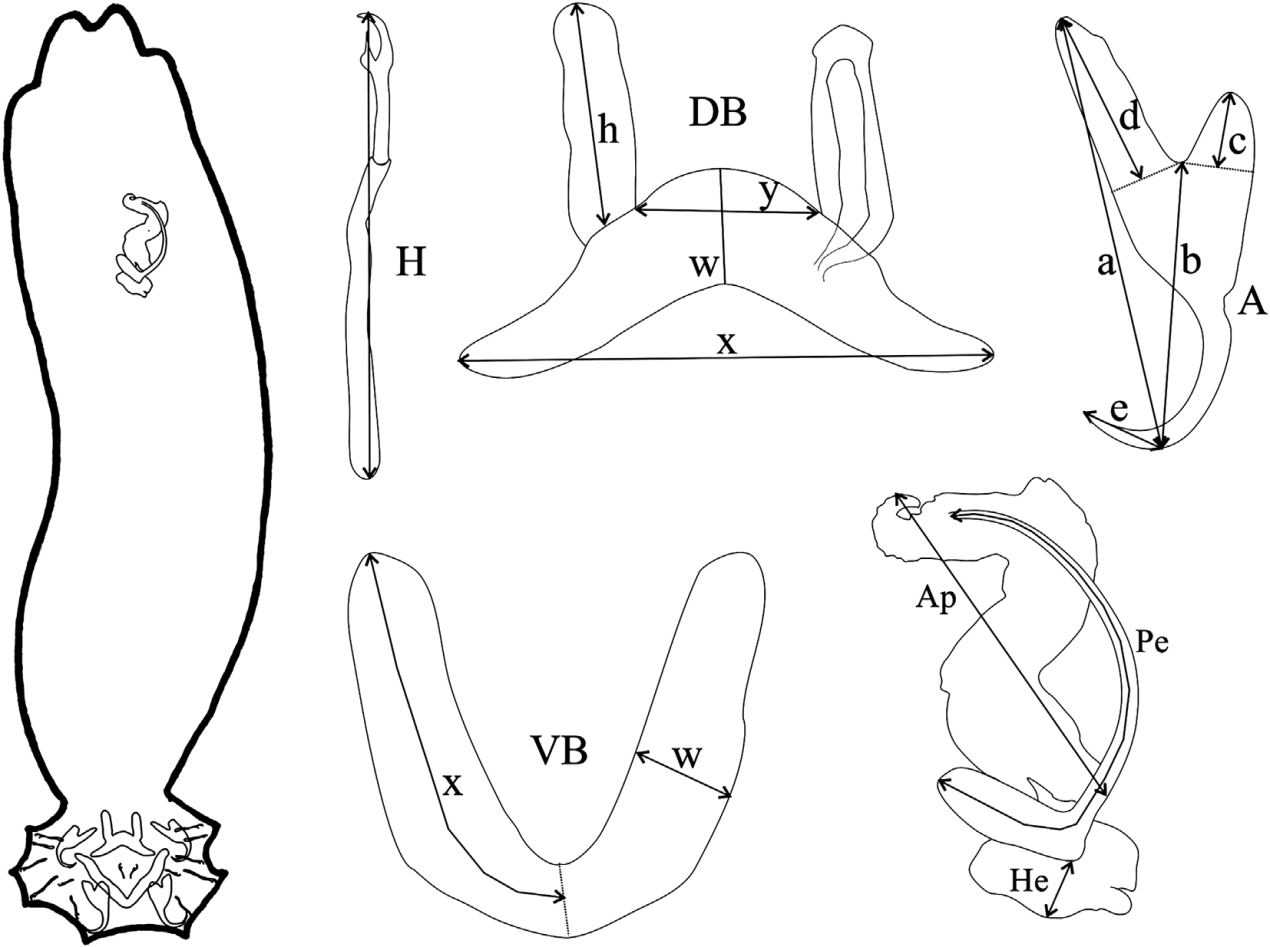
Schematic overview of a specimen of *Cichlidogyrus* with its typical morphological sclerotised structures (MCO, male copulatory organ in the upper part of the parasite image, attachment organ in the bottom part) and measurements used in the descriptions of the new species. Abbreviations: **A**, anchor (**a**, total length; **b**, blade length; **c**, shaft length; **d**, guard length; **e**, point length); **DB**, dorsal bar (**h**, auricle length; **w**, maximum straight width; **x**, total length; **y**, distance between auricles); **VB**, ventral bar (**x**, length of one ventral bar branch; **w**, maximum width); **H**, hook length; **Pe**, penis curved length; **He**, heel straight length; **Ap**, accessory piece straight length.

Five locations in southern Lake Victoria, Tanzania, were sampled (Table 1, Fig. 1, Table S1): Makobe rocky island (−2.3654, 32.9228) in May–August 2010 and in June–October 2014; Kissenda (−2.5494, 32.8276), Python (−2.6237, 32.8567), Luanso (−2.6889, 32.8842) rocky islands, and the swampy inlet stream Sweya (−2.5841, 32.8970) in June–October 2014. Fish were caught by angling and with gillnets of variable mesh sizes and morphologically identified by Ole Seehausen [51]. At Makobe, we collected 13 cichlid species belonging to the Lake Victoria radiation lineage and one species (*Astatoreochromis alluaudi*) representing an older lineage that did not speciate. At Sweya, we collected *A. alluaudi* as well as *Pseudocrenilabrus multicolor victoriae*, representing the other lineage that did not diversify, and *Astatotilapia nubila*, from the Lake Victoria radiation lineage. At Kissenda and Python, we collected three and one additional species of the Lake Victoria radiation lineage, respectively. Two of those (*Pundamilia* sp. ‘pundamilia-like’ and *Pundamilia* sp. ‘nyererei-like’) are closely related to *P. pundamilia* Seehausen & Bouton, 1998 and *P. nyererei* (Witte-Maas & Witte, 1985) that occur at Makobe. At Luanso, we collected one species (*Pundamilia* sp. ‘Luanso’). These species pairs of *Pundamilia* represent replicate speciation events [31]. The sampled cichlid species vary in their micro-habitat and trophic specialisations [2, 3, 18, 51, 65] and also in the extent of genetic differentiation [31, 32]. Within the radiation, divergence is 14,600 years old, while the divergence between both non-radiating lineages, and between them and the ancestors of the radiations in Lake Victoria, Lake Malawi and other lakes, is 8–10 million years old [31, 32, 48]. Fish were immediately sacrificed with an overdose of 2-phenoxyethanol, numbered and preserved in ethanol (some directly preserved in 100% ethanol, others fixed in 4% formaldehyde and then transferred to 70% ethanol). Sampling was conducted with permission from the Tanzania Commission for Science and Technology (COSTECH - No. 2013-253-NA-2014-117). In the laboratory, the right gill arches were removed and screened for macroparasites by inspecting gill filaments with a mounted needle, under a dissecting stereoscope (Zeiss Stemi 2000, Zeiss, Oberkochen, Baden-Württemberg, Germany). Monogeneans were detached with tweezers and individually stored in 100% ethanol. Specimens were individually mounted onto a slide, treated with 20% sodium dodecyl sulphate (SDS) to soften tissues and then fixed in Hoyer’s medium. Specimens of *Cichlidogyrus* were examined with a microscope (phase-contrast Olympus BX41TF, Olympus, Hamburg, Germany and Leica DM2500, Leica, Wetzlar, Hessen, Germany) and sclerotised parts (nomenclature and numbering according to ICOPA IV; [8]) were measured with Olympus Stream Essentials v. 1.9 and with LAS 6.0 software (Fig. 2, all measurements given in μm). Drawings of sclerotised parts were made with CorelDraw 2019 software (Corel Corporation, Ottawa, Ontario, Canada) on the basis of microphotographs taken with a Leica DM2500 microscope and LAS 6.0 software. Type material of *Cichlidogyrus longipenis* Paperna & Thurston, 1969 [38] (RMCA_VERMES_35921) and *C. bifurcatus* Paperna, 1960 [35] (RMCA_VERMES_35703) was measured and compared to our specimens. Type and voucher specimens of the parasites were deposited in the Muséum National d’Histoire Naturelle, Paris, France (MNHN), the Royal Museum for Central Africa, Tervuren, Belgium (RMCA) and the Iziko South African Museum, Cape Town, Republic of South Africa (SAMC); symbio(para)types and host vouchers (terminology: see [4]) are stored at the Swiss Federal Institute of Aquatic Science and Technology, Kastanienbaum, Switzerland (EAWAG). Photo vouchers are deposited on MorphoBank (https://morphobank.org/permalink/?P4937). For more details on the host-parasite combinations and geographical range of the species of *Cichlidogyrus* used for differential diagnosis, see Řehulková et al*.* [47] and Cruz-Laufer et al*.* [6]. Epidemiological indices (P = prevalence, IF = intensity range, MI = mean intensity) were calculated according to Bush et al*.* [5].

**Table 1:** Haplochromine host species (n fish: number of host individuals) sampled in May-August 2010 and June-October 2014 at five localities (Makobe, Sweya, Kissenda, Luanso, Python) in southern Lake Victoria and the sample size of identified specimens of *Cichlidogyrus* (n *Cichlidogyrus*)*.* The radiation lineage is labelled with a circle (●), the two lineages that did not speciate are labelled with a square (■) and a diamond (◆).

	Host sp.	Locality	n fish	n *Cichlidogyrus*
■	*Astatoreochromis alluaudi* Pellegrin, 1904	Makobe	9	106
■	*Astatoreochromis alluaudi* Pellegrin, 1904	Sweya	7	19
●	*Astatotilapia nubila* (Boulenger, 1906)	Sweya	15	6
●	*“Haplochromis” cyaneus* Seehausen, Bouton & Zwennes, 1998	Makobe	6	16
●	*Labrochromis* sp. ‘stone’	Makobe	11	3
●	*Mbipia lutea* Seehausen & Bouton, 1998	Makobe	5	14
●	*Mbipia mbipi* Seehausen, Lippitsch & Bouton 1998	Makobe	12	26
●	*Neochromis gigas* Seehausen & Lippitsch, 1998	Makobe	5	15
●	*Neochromis omnicaeruleus* Seehausen & Bouton, 1998	Makobe	16	58
●	*Neochromis rufocaudalis* Seehausen & Bouton, 1998	Makobe	7	13
●	*Neochromis* sp. ‘unicuspid scraper’	Makobe	33	46
●	*Paralabidochromis chilotes* Greenwood, 1959	Makobe	13	5
◆	*Pseudocrenilabrus multicolor victoriae* Seegers, 1990	Sweya	5	12
●	*Ptyochromis* sp. ‘striped rock sheller’	Makobe	4	3
●	*Ptyochromis xenognathus* Greenwood, 1957	Kissenda	11	18
●	*Pundamilia nyererei* (Witte-Maas & Witte, 1985)	Makobe	65	42
●	*Pundamilia pundamilia* Seehausen & Bouton, 1998	Makobe	62	50
●	*Pundamilia* sp. ‘Luanso’	Luanso	18	69
●	*Pundamilia* sp. ‘nyererei-like’	Kissenda	16	29
●	*Pundamilia* sp. ‘pink anal’	Makobe	14	21
●	*Pundamilia* sp. ‘pundamilia-like’	Kissenda	18	26
●	*Pundamilia* sp. ‘pundamilia-like’	Python	5	5

## Results

During this survey, eight species of Monopisthocotyla were found on the gills of studied hosts. With the exception of a single specimen of *Gyrodactylus sturmbaueri* infecting *Ptyochromis xenognathus* (Greenwood, 1957) from Kissenda (reported in [15] and deposited under accession number RMCA_VERMES_43410), all monogeneans corresponded to the diagnosis of *Cichlidogyrus* given in Pariselle and Euzet [40]. Two of the identified species of *Cichlidogyrus* were already known (*C. longipenis* Paperna & Thurston, 1969 [38], *C. bifurcatus* Paperna, 1960 [35]), the four others are herein formally described, and we characterise a potential fifth new species, for which we refrain from formal description for want of sufficient specimens.

### *Cichlidogyrus longipenis* Paperna & Thurston, 1969

*Previous records*: *Cichlidogyrus* sp. IV sensu Gobbin et al. [15, 16], see also [14]

*Type host*: *Astatoreochromis alluaudi* Pellegrin, 1904.

*Type locality*: Jinja, Uganda [38].

*Infection site*: Gills.

*Other records*: None.

*Current records*: Type host from Lake Victoria, Makobe Island and Sweya swampy inlet; *Pundamilia* sp. ‘pink anal’ from Lake Victoria, Makobe Island.

*Infection parameters*: Makobe: *Astatoreochromis alluaudi* P = 100% (9/9), IF = 2–17, MI = 10.7; *Pundamilia* sp. ‘pink anal’ P = 14.3% (2/14), IF = 1; Sweya: *A. alluaudi* P = 57.1% (4/7), IF = 1–14, MI = 4.5.

*Material studied*: 10 whole-mounted specimens in Hoyer’s solution and the syntype RMCA_VERMES_35921.

*Material deposited*: MNHN_HEL1476, MNHN_HEL1482, RMCA_VERMES_43419, SAMC-A092084.

*Voucher hosts*: *Astatoreochromis alluaudi* Pellegrin, 1904 from Makobe (EAWAG ID 103148 and 103567, Table S1).

**Figure 3:**
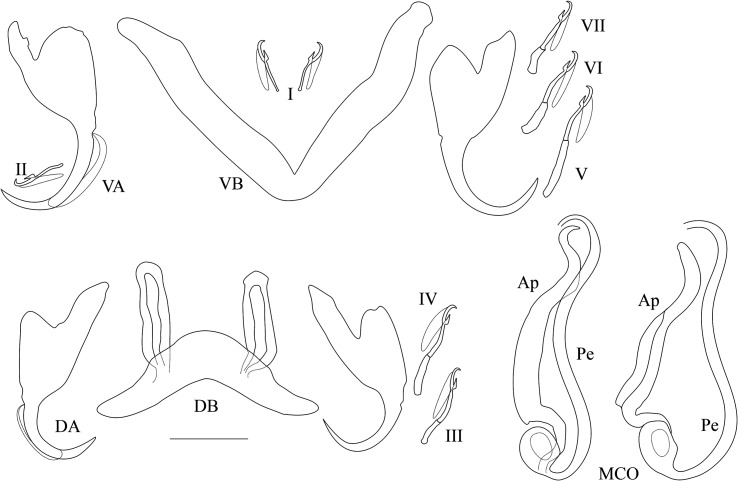
Sclerotised parts (haptor and male copulatory organ) of *Cichlidogyrus longipenis*. **I–VII**, hook pairs; **DA**, dorsal anchors; **DB**, dorsal transverse bar; **VA**, ventral anchors; **VB**, ventral transverse bar. **MCO**, male copulatory organ (on the left as observed in most of our specimens, on the right the phenotype similar to the one reported by Paperna & Thurston, 1969): **Ap**, accessory piece; **Pe**, penis. Scale bar: 20 μm.

*Redescription* (Table 2, Fig. 3): Two pairs of anchors of similar size and unequal shape (guard more developed in dorsal anchors). Ventral bar V-shaped. Dorsal bar with two auricles inserted at its dorsal surface. Hooks 7 pairs; I and III to VII short, except V of medium size (i.e., their total length relative to the total length of hook pairs II, see [39, 40]). Penis long, wavy, thinner at proximal and distal extremities, rounded basal bulb, no heel; simple accessory piece, attached to the basal bulb of the penis by a filament, ending in a curved hook. No sclerotised vagina.

**Table 2:** Measurements of *Cichlidogyrus bifurcatus* and *C. longipenis* redescribed from haplochromine host species of Lake Victoria (*N* = number of specimens, *n* = number of observations per trait per species) compared to those provided by Paperna [35] and Paperna & Thurston [38] (expressed as ranges only, because means were not reported in the original descriptions). For *C. longipenis*, we also report measurements extrapolated from the drawing of the original description and our measurements from the syntype. All measurements in μm, given as the mean and range (in parentheses).

		*C. longipenis* Paperna & Thurston, 1969					*C. bifurcatus* Paperna, 1960		
		Nobis		Paperna & Thurston 1969	Figure 6 in [38]	Syntype	Nobis		Paperna 1960
		*N* = 10		*N* = 3	*N* = 1	*N* = 1	*N* = 7		*N* = 7
Trait		Mean (min–max)	*n*	(min–max)			Mean (min–max)	*n*	
MCO	Ap	53.7 (43.9–60.6)	10	(29–40)	29.2	36.3	36.7 (34.3–39.5)	7	(30–47)
	He	1.5 (1.1–2.4)	6				6.6 (5.1–10.8)	7	
	Pe	98.7 (95.1–102.4)	10	(63–79)	60.2	72.3	47.7 (44.4–50.1)	7	(40–60)
DA	a	46.5 (39.2–53.5)	15	(23–33)	26.2	45.1	31.5 (29.4–34.1)	6	(30–40)
	b	32.4 (24.2–37.4)	15		21.2	25.6	20.8 (18.3–22.6)	6	(18–27)
	c	5.6 (4.6–7.3)	15		4.9	(6.2–7.3)	4.9 (3.7–6.2)	6	(4–19)
	d	15.7 (11.9–19.5)	15		12.9	(18.2–19.6)	12.3 (10.6–14.6)	6	(12–20)
	e	9.7 (7.7–12.3)	15		7.5	9.4	6.3 (5.9–6.7)	6	(5–10)
DB	h	20.7 (15.4–26.5)	19	(6–10)	11.2	13.4	13.8 (10.7–15.7)	6	(6–14)
	w	9.7 (7.9–12.0)	10		5.2	6.3	5.7 (5.4–6.0)	5	
	x	48.1 (38.4–55.6)	10	(23–33)	29.7	39.8	34.1 (32.3–38.7)	5	(25–36)
	y	15.9 (11.6–20.2)	10		9.1	13.5	13.2 (12.2–14.2)	5	
H	I	12.8 (12.0–13.7)	17	10			12.6 (10.1–14.2)	8	
	II	12.1 (10.3–13.1)	9	15	17.6	15.4	12.0 (11.0–13.3)	5	(12–17)
	III	17.8 (14.4–20.2)	10	24–27			18.3 (16.2–21.2)	5	
	IV	22.2 (18.7–24.8)	9				24.6 (22.2–26.3)	8	
	V	28.0 (25.8–32.6)	12				27.7 (26.0–30.0)	9	
	VI	22.8 (21.2–24.2)	9				24.7 (21.3–27.1)	4	
	VII	18.2 (16.0–19.3)	9				19.1 (17.6–20.3)	4	
VA	a	42.3 (35.3–48.2)	15	(20–38)	27.4	(37.4–39.2)	34.9 (31.5–37.7)	8	(30–40)
	b	38.2 (32.9–43.2)	15		21.8	(33.9–34.2)	27.0 (24.1–28.1)	8	(25–34)
	c	5.9 (2.9–9.6)	15		5.8	(4.6–4.8)	5.4 (3.4–7.2)	8	(5–11)
	d	10.7 (7.1–14.3)	15		10.7	12.2	10.9 (8.3–13.9)	9	(7–13)
	e	12.0 (9.1–13.8)	15		8.3	(11.2–11.4)	8.5 (7.7–9.1)	6	(5–10)
VB	w	8.7 (7.0–11.4)	20		5.4	4.9	5.7 (5.3–6.1)	5	
	x	50.7 (40.3–62.1)	20	(53–73)	29.2	38.6	36.3 (29.6–40.4)	10	(40–70)

**Figure 4:**
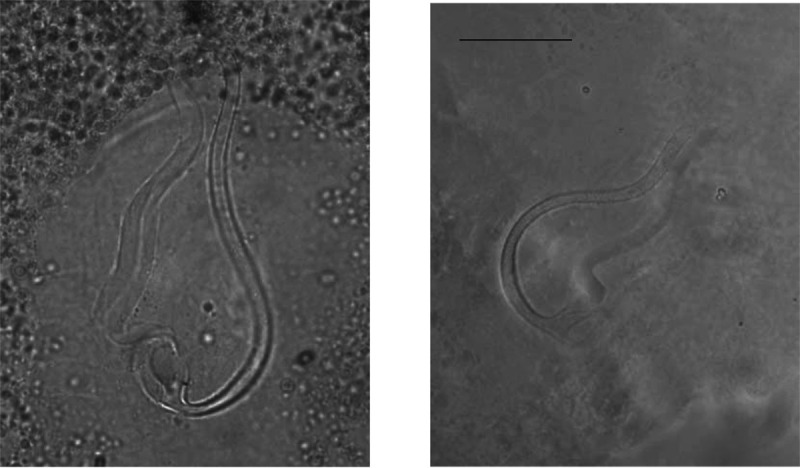
Micrographs of the male copulatory organ of *Cichlidogyrus longipenis*, from a parasite individual of the present study (**left**) and from the holotype by Paperna & Thurston, 1969 (**right**), fixed in two different mediums. Scale bar: 20 μm.

*Remarks*: The specimens described herein share the combination of the following characters only with *C. longipenis* [38]: hook pairs I and II to VII short (or of medium size), penis long (>60 μm) with simple accessory piece and no heel, no sclerotised vagina. The measurements of some of these characters differ between the original description and our data (Table 2). Some traits were measured as larger when compared to values reported in the original description: the length of the accessory piece (43.9–60.6 vs. 29–40 μm, respectively), penis (95.1–102.4 vs. 63–79 μm), ventral (35.3–48.2 vs. 20–38 μm) and dorsal anchors (39.2–53.5 vs. 23–33 μm), auricles (15.4–26.5 vs. 6–10 μm), dorsal bar (38.4–55.6 vs. 23–33 μm), ventral bar (40.3–62.1 vs. 53–73 μm). Even so, we are confident of our identification of these specimens as *C. longipenis*: as mentioned above, species identification in *Cichlidogyrus* is mainly based on the morphology of the reproductive apparatus [43, 61], which is similar in size and shape in our specimens compared to the type (Figs. 3 and 4). The observed size differences may be due to two non-exclusive factors. First, the mounting medium used: Fankoua et al*.* [9] demonstrated that the use of Hoyer’s medium increases the size and modifies the shape of sclerotized parts. Second, the way Paperna and Thurston [38] made the drawings and took the measurements for the original description: although measurements given in the original description are compatible with its drawings and scale bars (Table 2), there are significant differences between these measurements and those we took from the syntype (Table 2) (an exception is the ventral bar total length, which seems incomparable: we measured the length of a branch, while the original authors probably measured the total length of the bar). This is probably because Paperna and Thurston [38] did not use a camera lucida and made their drawings freehand, which may have led to magnification errors.

### *Cichlidogyrus bifurcatus* Paperna, 1960 [35]

*Previous records*: *Cichlidogyrus* sp. VI sensu Gobbin et al. [15, 16], see also [14].

*Type host*: *Astatotilapia flaviijosephi* (Lortet, 1883).

*Type locality*: Sea of Galilee (spelled Sea of Gallilee in the original description) [35].

*Infection site*: Gills.

*Other previous records*: Young *Oreochromis aureus* (L.) from the Sea of Galilee (initially identified as *O. niloticus*) [34]. *Harpagochromis squamipinnis* Regan, 1921 and “*Haplochromis”* sp. ‘aeneocolor’ Greenwood, 1973 from Lake George, and Kazinga channel; “*H.” elegans* Trewavas, 1933 and *Haplochromis limax* Trewavas, 1933 from Lake George; “*Haplochromis”* sp. from Lake Edward and Entebbe, Lake Victoria; *Pseudocrenilabrus multicolor* (Schöller, 1903) from Lake Mulehe and a stream near Masindi, Lake Albert system, Uganda [37].

*Current records*: *Pseudocrenilabrus multicolor victoriae* Seegers, 1990 and “*Astatotilapia” nubila* (Boulenger, 1906) from Lake Victoria, Sweya swampy inlet stream*.*

*Infection parameters*: *Pseudocrenilabrus multicolor victoriae* P = 11.5% (3/26), IF = 2; “*Astatotilapia” nubila* P = 6.7% (1/15), IF = 1.

*Material studied*: 7 whole-mounted specimens in Hoyer’s solution and the paratype RMCA_VERMES_35.703.

*Material deposited*: MNHN_HEL1470.

*Voucher host*: *Pseudocrenilabrus multicolor victoriae* Seegers, 1990 from Sweya (EAWAG ID 109436, Table S1).

**Figure 5:**
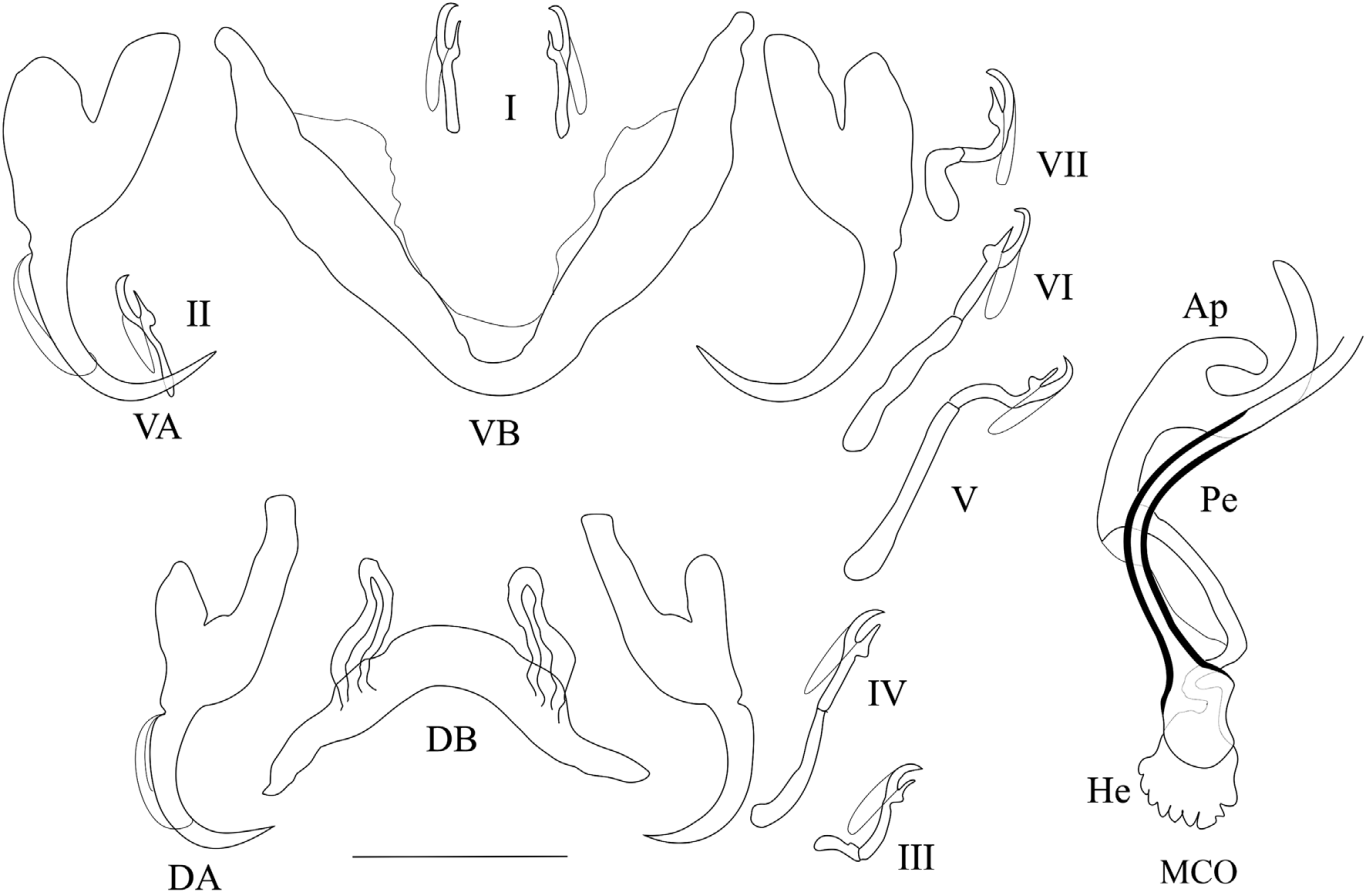
Sclerotised parts (haptor and male copulatory organ) of *Cichlidogyrus bifurcatus*. **I–VII**, hook pairs; **DA**, dorsal anchors; **DB**, dorsal transverse bar, **VA**, ventral anchors; **VB**, ventral transverse bar. **MCO**, male copulatory organ: **AP**, accessory piece; **Pe**, penis; **He**, heel. Scale bar: 20 μm.

*Redescription* (Table 2, Fig. 5): Two pairs of anchors of similar size, with guard approximately 2 times as long as shaft. Dorsal anchors with guard and shaft more asymmetrical and blade shorter than ventral anchors. Ventral bar V-shaped, with 2 branches with wing-shaped attachments along distal half. Dorsal bar thin, tapering towards its extremities, and 2 small auricles inserted at its dorsal surface. Hooks 7 pairs; I short (i.e., its total length less than 1.7 times the total length of hook pair II); III to VII short (i.e., their total length less than 2 times the total length of hook pair II), V and VI longer (see [39, 40]). Male copulatory organ (MCO) consisting of slightly curved penis with large basal bulb and constant diameter; accessory piece simple, terminally frayed; developed heel with crenelated distal edge. Vagina not sclerotised.

*Remarks*: The specimens found in Lake Victoria on *P. multicolor victoriae* and “*A.” nubila*, identified as *C. bifurcatus*, display many similarities and some dissimilarities with *C. bifurcatus* described by Paperna in 1960 on *A. flaviijosephi* in the Sea of Galilee. They are similar in the shape and size of the MCO, the hooks, anchors and bars. Only some range extensions and minor differences in the size of some sclerotised parts are apparent, e.g., the length of the ventral bar (see Table 2), that may be due to two reasons, see remarks above under *C. longipenis*.

### *Cichlidogyrus pseudodossoui* n. sp.

urn:lsid:zoobank.org:act:2AA5987B-7AE1-407E-8E58-CB6CEFC89360

*Previous records*: *Cichlidogyrus* sp. III sensu Gobbin et al. [15, 16], *C.* sp. “pseudodossoui” sensu Gobbin et al. [17], see also [14].

*Type host*: *Astatoreochromis alluaudi* Pellegrin, 1904.

*Type locality*: Off Makobe Island, Lake Victoria.

*Holotype*: MNHN_HEL1478.

*Paratypes*: MNHN_HEL1473, RMCA_VERMES_43421, SAMC-A092086.

*Infection site*: Gills.

*Other hosts*: *Neochromis omnicaeruleus* Seehausen & Bouton, 1998; *Neochromis* sp. ‘unicuspid scraper’; *Ptyochromis xenognathus* (Greenwood, 1957); *Pundamilia nyererei* (Witte-Maas & Witte, 1985); *Pundamilia* sp. ‘nyererei-like’; *Pundamilia pundamilia* Seehausen & Bouton, 1998; *Pundamilia* sp. ‘pundamilia-like’.

*Other localities*: Kissenda Island, Python Island, Sweya swampy inlet.

*Infection parameters*: Makobe: *Astatoreochromis alluaudi* P = 55.5% (5/9), IF = 1–2, MI = 1.2; *Neochromis omnicaeruleus* P = 6.2% (1/16), IF = 1; *Neochromis* sp. ‘unicuspid scraper’ P = 3.1% (1/32), IF = 1; *Pundamilia pundamilia* P = 1.6% (1/61), IF = 1; *P. nyererei* P = 4.6% (3/65), IF = 1–2, MI = 1.6; Kissenda: *Ptyochromis xenognathus* P = 9.1% (1/11), IF = 1; *Pundamilia* sp. *‘*pundamilia-like’ P = 5.6% (1/18), IF = 1; *Pundamilia* sp. ‘nyererei-like’ P = 6.2% (1/16), IF = 2; Python: *Pundamilia* sp. ‘pundamilia-like’ P = 20% (1/5), IF = 2; Sweya: *A. alluaudi* P = 14.3% (1/7), IF = 1.

*Material studied*: 7 whole-mounted specimens in Hoyer’s solution.

*Symbiotype*: *Astatoreochromis alluaudi* Pellegrin, 1904 from Makobe (EAWAG ID 103148, Table S1).

*Symbioparatypes*: *Astatoreochromis alluaudi* Pellegrin, 1904 from Makobe (EAWAG ID 103571, Table S1); *Neochromis omnicaeruleus* Seehausen & Bouton, 1998 from Makobe (EAWAG ID 105655, Table S1); *Ptyochromis xenognathus* (Greenwood, 1957) from Kissenda (EAWAG ID 12306, Table S1).

*Etymology*: The species epithet refers to the similarity of this newly described species with its congener *C. dossoui* Douëllou, 1993.

**Figure 6:**
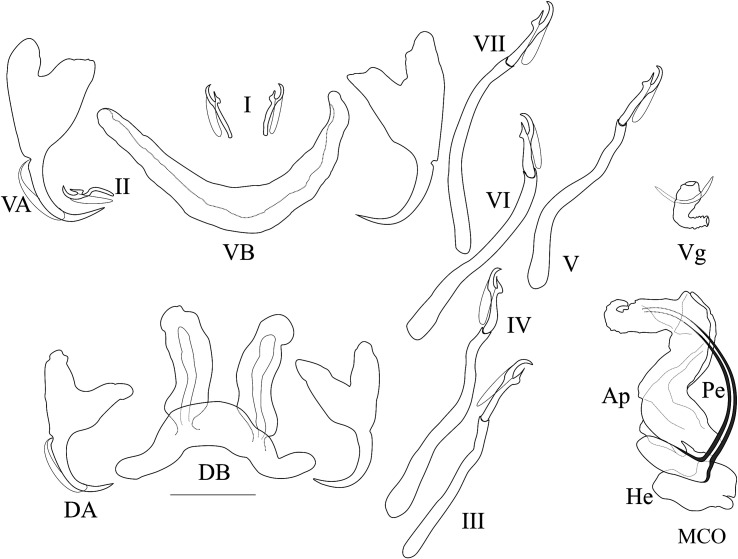
Sclerotised parts (haptor and male copulatory organ) of *Cichlidogyrus pseudodossoui* n. sp. **I–VII**, hook pairs; **DA**, dorsal anchors; **DB**, dorsal transverse bar; **VA**, ventral anchors; **VB**, ventral transverse bar. **MCO**, male copulatory organ: **AP**, accessory piece; **Pe**, penis; **He**, heel; **Vg**, vagina. Scale bar: 20 μm.

*Description* (Table 3, Fig. 6): Two pairs of anchors of unequal size (ventral larger) and similar shape: short blade, and shaft and guard symmetrical in appearance and of similar size. Ventral bar V-shaped (rounded on its anterior edge). Dorsal bar with two developed auricles inserted at its dorsal surface. Hooks 7 pairs; I short; III to VII very long (see [39, 40]). MCO consisting of a J-shaped and thin penis starting at right angle from an ovoid bulb; accessory piece thick, Z-shaped, with a recurved tooth at its distal extremity; developed rounded heel. Vagina thick walled, bent at right angle, with annulated proximal third, broadened towards distal extremity.

**Table 3:** Measurements of the new species of *Cichlidogyrus* described from haplochromine host species of Lake Victoria (*N* = number of specimens, *n* = number of observations per trait per species). All measurements in μm, given as the mean and range (in parentheses).

		*C. pseudodossoui* n. sp.		*C. nyanza* n. sp.		*C.* cf. *furu*		*C. furu* n. sp.		*C. vetusmolendarius* n. sp.	
N		*N* = 7		*N* = 11		*N* = 1		*N* = 10		*N* = 10	
Trait		Mean (min-max)	*n*	Mean (min-max)	*n*	Mean (min-max)	*n*	Mean (min-max)	*n*	Mean (min-max)	*n*
MCO	Ap	41.3 (35.3–55.2)	7	40.2 (31.2–50.2)	11	36.5	1	43.3 (30.0–47.7)	10	23.0 (18.8–26.2)	6
	He	9.2 (8.5–10.7)	7	0.0 (0.0–0.0)	0	4.8	1	7.4 (6.5–7.9)	10	4.5 (3.0–5.2)	6
	Pe	63.4 (57.3–69.1)	7	45.9 (42.4–52.6)	11	58.6	1	58.4 (52.9–69.5)	10	32.6 (28.4–34.9)	6
DA	a	33.6 (28.2–38.7)	5	35.8 (31.9–38.5)	17	37.6	1	33.1 (28.1–37.1)	17	38.8 (34.5–41.2)	10
	b	25.0 (21.4–27.6)	5	28.6 (25.9–31.7)	17	26.1	1	22.1 (19.6–24.9)	17	24.0 (20.9–27.0)	10
	c	13.9 (9.6–17.0)	6	4.7 (3.4–8.8)	17	4.8	1	6.4 (4.7–8.3)	17	5.9 (3.9–7.9)	10
	d	12.4 (7.8–14.7)	6	10.8 (7.6–14.2)	17	14.4	1	14.1 (11.8–18.1)	17	16.9 (13.4–19.6)	10
	e	7.6 (6.3–9.2)	6	9.1 (7.2–10.5)	17	7.0	1	7.6 (6.2–9.2)	15	9.3 (7.7–10.8)	11
DB	h	28.8 (22.5–38.6)	11	22.1 (19.1–28.3)	20	16.7 (16.5-16.9)	2	18.6 (13.7–21.3)	19	17.2 (13.0–20.2)	18
	w	8.3 (6.8–9.3)	7	8.6 (5.7–10.9)	11	7.6	1	7.7 (5.6–8.8)	10	7.3 (5.6–8.9)	9
	x	49.8 (42.0–61.2)	7	51.9 (39.9–59.1)	11	32.4	1	40.1 (35.1–43.5)	10	44.0 (35.3–49.0)	9
	y	8.7 (5.7–12.8)	7	14.4 (10.1–17.3)	11	13.0	1	12.1 (8.9–14.5)	10	13.9 (10.6–18.5)	9
H	I	12.2 (10.5–15.0)	7	13.5 (10.8–15.0)	14	11.6	1	12.3 (11.2–13.5)	19	30.7 (26.3–35.3)	17
	II	13.3 (10.7–15.2)	8	12.3 (11.1–13.6)	14			11.8 (11.1–12.8)	15	11.9 (10.3–13.8)	10
	III	58.0 (45.5–69.8)	8	16.8 (15.0–18.8)	14	18.6	1	15.3 (13.9–15.9)	12	22.8 (18.9–25.7)	9
	IV	62.9 (43.7–74.5)	10	20.2 (17.9–22.7)	14	19.7	1	22.7 (19.3–25.3)	13	27.3 (24.6–29.4)	12
	V	70.0 (50.6–78.5)	8	21.8 (20.3–24.6)	12	22.3	1	24.5 (21.0–27.3)	14	31.0 (28.6–35.1)	5
	VI	66.0 (49.9–77.6)	9	18.8 (16.3–20.5)	15	16.2	1	19.2 (16.6–20.9)	16	28.0 (25.6–33.1)	8
	VII	62.3 (48.7–76.1)	9	17.4 (15.8–19.0)	14	13.7	1	16.4 (14.5–17.6)	17	23.2 (19.2–25.5)	6
VA	a	38.0 (33.7–44.2)	10	35.1 (31.5–41.8)	19	35.8	1	32.7 (30.1–36.2)	17	34.0 (29.1–41.3)	16
	b	32.3 (27.1–37.6)	10	32.4 (30.4–35.2)	19	31.9	1	27.7 (24.3–29.8)	17	29.8 (26.6–33.1)	16
	c	9.9 (5.9–15.3)	10	5.9 (3.7–8.8)	18	6.4	1	5.9 (3.9–10.0)	17	5.0 (2.5–6.7)	15
	d	12.9 (7.9–18.4)	10	10.5 (7.6–13.9)	19	11.7	1	10.7 (7.3–14.1)	17	10.6 (7.4–14.5)	15
	e	9.4 (8.8–11.0)	10	11.1 (7.5–12.6)	18	7.6	1	10.1 (8.6–11.8)	16	11.8 (9.5–13.9)	15
VB	w	8.5 (6.7–11.4)	7	10.0 (6.8–12.4)	13	6.5	1	7.0 (5.4–8.4)	10	6.3 (4.7–7.8)	9
	x	38.9 (32.0–45.1)	14	43.8 (36.9–50.6)	22	40.7	1	41.4 (36.4–45.2)	20	50.5 (37.0–61.2)	16
Vg	L	13.1 (11.9–15.1)	5								
	l	5.1 (4.2–6.1)	5								

*Remarks*: This new species belongs to the group showing the following characters: hooks I short, long hooks III–VII, penis J-shaped, accessory piece Z-shaped without auxiliary plate, and sclerotised vagina. This group also comprises the following 15 species: *C. anthemocolpos* Dossou, 1982 (on a few coptodonine species in West Africa), *C. bonhommei* Pariselle & Euzet, 1998 (on heterotilapiine species in West Africa), *C. bouvii* Pariselle & Euzet, 1997 (on an oreochromine species in West Africa), *C. dossoui* Douëllou, 1993 (on several coptodonine species, a haplochromine, a pelmatolapiine, a tilapiine species in Central and West Africa, and introduced with oreochromines elsewhere), *C. douellouae* Pariselle, Bilong Bilong & Euzet, 2003 (on an oreochromine in West Africa), *C. ergensi* Dossou, 1982 (on several coptodonine species, a pelmatolapiine species in West Africa and the Middle East), *C. flexicolpos* Pariselle & Euzet, 1995 (on several coptodonine species and a pelmatolapiine species in West Africa), *C. gillesi* Pariselle, Bitja Nyom & Bilong Bilong, 2013 (on a coptodonine species in Central Africa), *C. hemi* Pariselle & Euzet, 1998 (on a tilapiine species in West Africa), *C. kouassii* N’Douba, Thys van den Audenaerde & Pariselle, 1997 (on a coptodonine species in Southern-West Africa), *C. legendrei* Pariselle & Euzet, 2003 (on a pelmatolapiine species in Central Africa), *C. lemoallei* Pariselle & Euzet, 2003 (on a few pelmatolapiine species in Central Africa), *C. ouedraogoi* Pariselle & Euzet, 1996 (on a few coptodonine and a pelmatolapiine species in Central and West Africa), *C. tiberianus* Paperna, 1960 (on several coptodonine and oreochromine species, a few haplochromine and tilapiine species, a pelmatolapiine species in Central and West Africa and in the Middle East), and *C. vexus* Pariselle & Euzet, 1995 (on a few coptodonine species in West Africa). *Cichlidogyrus pseudodossoui* n. sp. mainly differs by the shape and length of its vagina (short, conical, and bent at right angle) from *C. flexicolpos*, *C. lemoallei* (vagina very long, thin and folded back in the middle); from *C. ergensi*, *C. gillesi*, *C. kouassii*, and *C. ouedraogoi* (vagina S-shaped); from *C. anthemocolpos* (U-shaped with a distal plate), *C. bonhommei* (thin walled, bent in two different perpendicular plans), *C. hemi* (straight, with constant diameter and annulated all along), *C. legendrei* (well developed), *C. tiberianus* (looped, 1 turn). *Cichlidogyrus pseudodossoui* n. sp. resembles *C. dossoui* in the shape of the accessory piece with a recurved distal end, the J-shaped thin penis starting in an ovoid bulb, and the rounded heel of the MCO. It is close to *C. bouvii*, *C. dossoui*, *C. douellouae*, and *C. vexus*, which all have a conically shaped vagina bending at a right angle. It differs from these species by the greater length of its dorsal bar auricles (which have a size range of 22.5–38.6 μm in *C. pseudodossoui* n. sp. vs. 10–15, 12–20, 11–17, 13–20 μm, respectively for the four other species) and above all of its hook pairs III–VII. These have a size range of 45.5–78.5 μm in *C. pseudodossoui* n. sp. vs. 31–43, 27–37, 31–43 μm, respectively for *C. bouvii*, *C. douellouae*, and *C. vexus*. These hook lengths also allow us to distinguish between *C. dossoui* and *C. pseudodossoui* n. sp. as follows: III: 36–45 vs. 45.5–69.8, IV: 38–50 vs. 43.7–74.5, V: 40–50 vs. 50.6–78.5, VI: 40–49 vs. 49.9–77.6, VII: 36–48 vs. 48.8–76.1.

### *Cichlidogyrus nyanza* n. sp.

urn:lsid:zoobank.org:act:3CCDC28A-959D-4336-B752-951B0F0DFCC7

*Previous records*: *Cichlidogyrus* sp. I sensu Gobbin et al. [15, 16], *C.* sp. “nyanza” sensu Gobbin et al. [17], see also [14].

*Type host*: “*Haplochromis” cyaneus* Seehausen, Bouton & Zwennes, 1998.

*Type locality*: Off Makobe Island, Lake Victoria.

*Holotype*: MNHN_HEL1477.

*Paratypes*: MNHN_HEL1472, RMCA_VERMES_43420, SAMC-A092085.

*Infection site*: Gills.

*Other hosts*: *“Haplochromis” cyaneus* Seehausen, Bouton & Zwennes, 1998; *Labrochromis* sp. ‘stone’; *Mbipia lutea* Seehausen & Bouton, 1998; *M. mbipi* Seehausen, Lippitsch & Bouton, 1998; *Neochromis gigas* Seehausen & Lippitsch 1998; *N. omnicaeruleus* Seehausen & Bouton, 1998; *N. rufocaudalis* Seehausen & Bouton, 1998; *Neochromis* sp. ‘unicuspid scraper’; *Paralabidochromis chilotes* Greenwood, 1959; *Ptyochromis xenognathus* Greenwood, 1957; *Ptyochromis* sp. ‘striped rock sheller’; *Pundamilia* Seehausen & Bouton, 1998; *Pundamilia* sp. ‘pundamilia-like’; *P. nyererei* (Witte-Maas & Witte, 1985); *Pundamilia* sp. ‘nyererei-like’; *Pundamilia* sp. ‘Luanso’; *Pundamilia* sp. ‘pink anal’; *Pseudocrenilabrus multicolor victoriae* Seegers, 1990.

*Other localities*: Kissenda Island, Python Island, Luanso Island, Sweya swampy inlet.

*Infection parameters*: Makobe: *“Haplochromis” cyaneus* P = 83.3% (5/6), IF = 1–5, MI = 3.0; *Labrochromis* sp. ‘stone’ P = 9.1% (1/11), IF = 1–2, MI = 1.5; *Mbipia lutea* P = 60% (3/5), IF = 3–5, MI = 4.0; *M. mbipi* P = 66.6% (8/12), IF= 1–3, MI = 1.6; *Neochromis gigas* P = 600% (3/5), IF = 4–6, MI = 5.0; *N. omnicaeruleus* P = 75.0% (12/16), IF = 1–17, MI = 4.1, *N. rufocaudalis* P = 57.1% (4/7), IF = 1–4, MI = 2.7; *Neochromis* sp. ‘unicuspid scraper’ P = 59.4% (19/32), IF = 1–6, MI = 2.0; *Paralabidochromis chilotes* P = 30.7% (4/13), IF = 1; *Ptyochromis* sp. ‘striped rock sheller’ P = 25% (1/4), IF = 3; *Pundamilia nyererei* P = 7.7% (5/65), IF = 1–3, MI = 1.8; *Pundamilia* sp. ‘pink anal’ P = 35.7% (5/14), IF = 1–5, MI = 1.8; *P. pundamilia* P = 24.2% (15/62), IF = 1–6, MI = 2.4; Kissenda: *Ptyochromis xenognathus* P = 36.4% (4/11), IF = 1–5, MI = 3.5; *Pundamilia* sp. ‘nyererei-like’ P = 50.0% (8/16), IF = 1–2, MI = 1.4; *Pundamilia* sp. ‘pundamilia-like’ P = 50.0% (9/18), IF = 1–3, MI = 1.6; Python: *Pundamilia* sp. ‘pundamilia-like’ P = 20% (1/5), IF = 3; Luanso: *Pundamilia* sp. ‘Luanso’ P = 27.7% (5/18), IF = 1–3, MI = 1.6; Sweya: *Pseudocrenilabrus multicolor victoriae* P = 3.8% (1/26), IF = 1.

*Material studied*: 11 whole-mounted specimens in Hoyer’s solution.

*Symbiotype*: *“Haplochromis” cyaneus* Seehausen, Bouton & Zwennes, 1998 from Makobe (EAWAG ID 105317, Table S1).

*Symbioparatypes*: “*Haplochromis” cyaneus* Seehausen, Bouton & Zwennes, 1998 from Makobe (EAWAG ID 105323 and 105128, Table S1).

*Etymology*: The species epithet, a noun in apposition, refers to the word for lake in several regional languages of East Africa.

**Figure 7:**
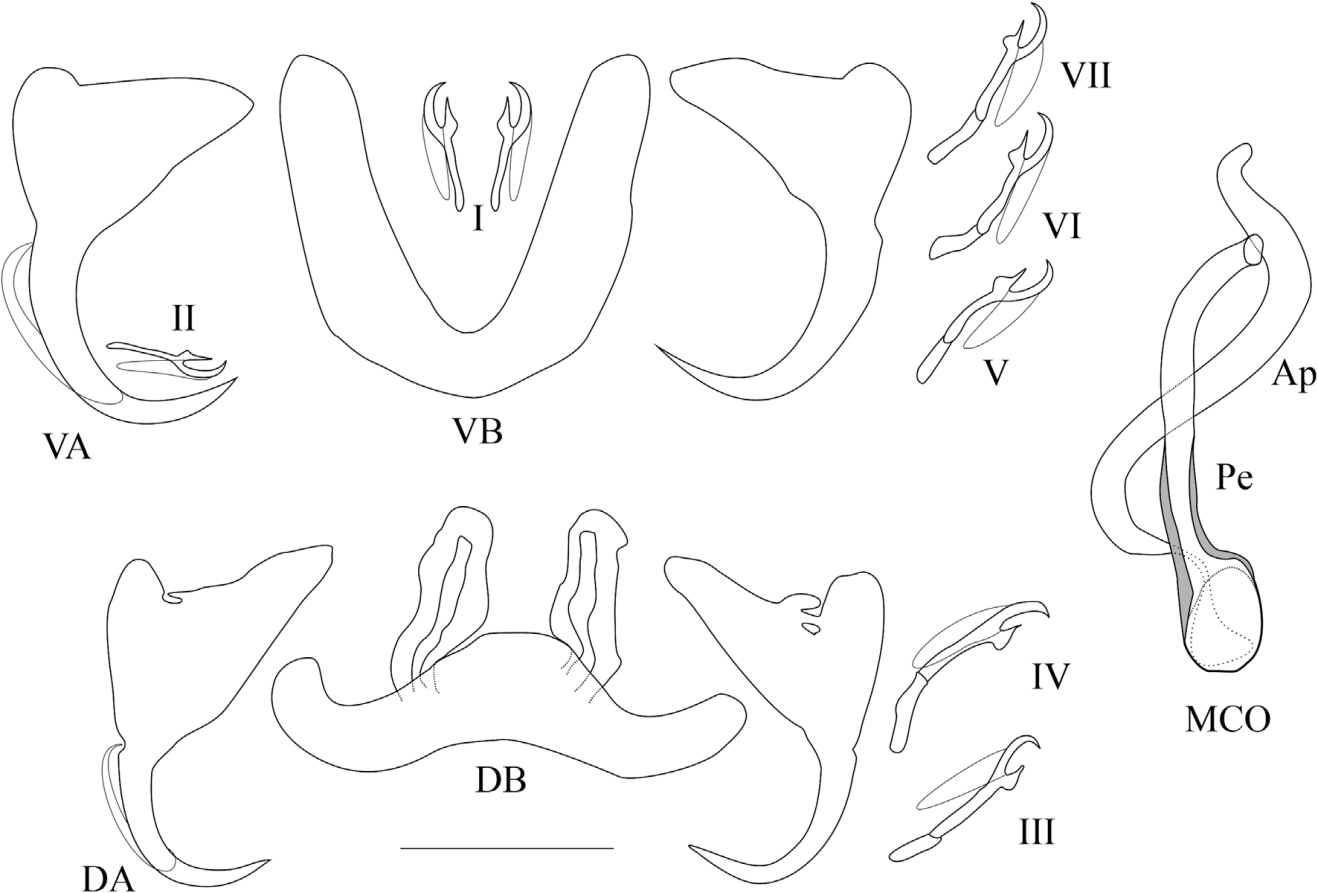
Sclerotised parts (haptor and male copulatory organ) of *Cichlidogyrus nyanza* n. sp*.* **I–VII**, hook pairs; **DA**, dorsal anchors; **DB**, dorsal transverse bar; **VA**, ventral anchors; **VB**, ventral transverse bar. **MCO**, male copulatory organ: **AP**, accessory piece; **Pe**, penis. Scale bar: 20 μm.

*Description* (Table 3, Fig. 7): Two pairs of anchors of similar size and shape, with short shaft, dorsal anchor sometimes with fenestrations at the junction of shaft and guard. Ventral bar U-shaped with thick branches. Dorsal bar with two auricles inserted at its dorsal surface. Hooks 7 pairs; I and III to VII short (see [39, 40]). MCO consisting of a large penis with constant diameter but thicker wall at its basis, bent at distal third, accessory piece simple, S-shaped, attached to the basal bulb of the penis by a thin filament; no heel. No sclerotised vagina.

*Remarks*: Short hook pairs I and III–VII, a simple MCO (see description above), and the absence of a sclerotised vagina are features that *Cichlidogyrus nyanza* n. sp. shares with several congeners infecting haplochromine cichlids in Central and Southern-East Africa, such as *C. banyankimbonai* Pariselle & Vanhove, 2015; *C. frankwillemsi* Pariselle & Vanhove, 2015; *C. franswittei* Pariselle & Vanhove, 2015; *C. georgesmertensi* Pariselle & Vanhove, 2015; *C. gillardinae* Muterezi Bukinga, Vanhove, Van Steenberge & Pariselle, 2012; *C. gistelincki* Gillardin, Vanhove, Pariselle, Huyse & Volckaert, 2012; *C. irenae* Gillardin, Vanhove, Pariselle, Huyse & Volckaert, 2012; *C. muterezii* Pariselle & Vanhove 2015; *C. raeymaekersi* Pariselle & Vanhove, 2015, and *C. steenbergei* Gillardin, Vanhove, Pariselle, Huyse & Volckaert, 2012. The new species described herein differs from all of these in showing both an accessory piece reaching beyond the distal end of the penis and no heel. *Cichlidogyrus haplochromii* and *C. tilapiae*, previously reported from Lake Victoria (on several haplochromine and a few oreochromine species) also display short hooks and a simple MCO, but have no heel, such as in *C. nyanza* n. sp., which differs from them in the shape of the accessory piece (of which the terminal end is blunter in *C. nyanza* n. sp. than the hook-like extension of *C. haplochromii* Paperna & Thurston, 1969 or the pointed extremity in *C. tilapiae* Paperna, 1960) and the shape of the ventral anchors (less incised in *C. nyanza* n. sp.).

### *Cichlidogyrus furu* n. sp.

urn:lsid:zoobank.org:act:C1E2FD2F-0BB1-4154-8C96-552C261283CC

*Previous records*: *Cichlidogyrus* sp. II sensu Gobbin et al. [15, 16], *C.* sp. “furu” sensu Gobbin et al. [17], see also [14].

*Type host*: *Pundamilia nyererei* (Witte-Maas & Witte, 1985).

*Type locality*: Off Makobe Island, Lake Victoria.

*Holotype*: MNHN_HEL1475.

*Paratypes*: MNHN_HEL1471, RMCA_VERMES_43418.

*Infection site*: Gills.

*Other hosts*: *Astatoreochromis alluaudi* Pellegrin, 1904; “*Astatotilapia” nubila* (Boulenger, 1906); *“Haplochromis” cyaneus* Seehausen, Bouton & Zwennes, 1998; *Mbipia lutea* Seehausen & Bouton, 1998; *M. mbipi* Seehausen, Lippitsch & Bouton, 1998; *Neochromis omnicaeruleus* Seehausen & Bouton, 1998; *N. rufocaudalis* Seehausen & Bouton 1998; *Neochromis* sp. ‘unicuspid scraper’; *Paralabidochromis chilotes* Greenwood, 1959; *Pseudocrenilabrus multicolor victoriae* Seegers, 1990; *Ptyochromis xenognathus* Greenwood, 1957; *Pundamilia* sp. ‘Luanso’; *Pundamilia nyererei* (Witte-Maas & Witte, 1985); *Pundamilia* sp. ‘pink anal’; *P. pundamilia* Seehausen & Bouton, 1998; *Pundamilia* sp. ‘nyererei-like’ and *Pundamilia* sp. ‘pundamilia-like’ from Kissenda.

*Other localities*: Kissenda Island, Luanso Island, Sweya swampy inlet.

*Infection parameters*: Makobe: *Astatoreochromis alluaudi* P = 22.2% (2/9), IF = 1–2, MI = 1.5; *“Haplochromis” cyaneus* P = 16.7% (1/6), IF = 1; *Mbipia lutea* P = 20.0% (1/5), IF = 1; *M. mbipi* P = 75.0% (9/12), IF = 1–3, MI = 1.4; *Neochromis omnicaeruleus* P = 31.2% (5/16), IF = 1–3, MI = 1.6; *N. rufocaudalis* P =28.6% (2/7), IF = 1; *Neochromis* sp. ‘unicuspid scraper’ P = 18.2% (6/33), IF = 1; *Paralabidochromis chilotes* P = 7.7% (1/13), IF = 1; *Pundamilia pundamilia* P = 11.3% (7/62), IF = 1–3, MI = 1.6; *P. nyererei* P = 23.1% (15/65), IF = 1–5, MI = 1.6; *Pundamilia* sp. ‘pink anal’ P = 21.4% (3/14), IF = 1–5; MI = 2.6; Kissenda: *Ptyochromis xenognathus* P = 18.2% (2/11), IF = 1–2, MI = 1.5; *Pundamilia* sp. ‘nyererei-like’ P = 43.7% (7/16), IF = 1–3, MI = 1.6; *Pundamilia* sp. ‘pundamilia-like’ P = 33.3% (6/18), IF = 1–3, MI = 1.5; Luanso: *Pundamilia* sp. ‘Luanso’ P = 44.4% (8/18), IF = 1–5, MI = 2.1; Sweya: “*Astatotilapia” nubila* P = 6.7% (1/15), IF = 3; *Pseudocrenilabrus multicolor victoriae* P = 7.7% (2/26), IF = 1.

*Material studied*: 10 whole-mounted specimens in Hoyer’s solution.

*Symbiotype*: *Pundamilia nyererei* (Witte-Maas & Witte, 1985) from Makobe (EAWAG ID 103397, Table S1).

*Symbioparatypes*: *Pundamilia nyererei* (Witte-Maas & Witte, 1985) from Makobe Island (EAWAG ID 103312 and 103397, Table S1).

*Etymology*: The species epithet is the word referring to haplochromine cichlids in Kiswahili, used as a noun in apposition.

**Figure 8:**
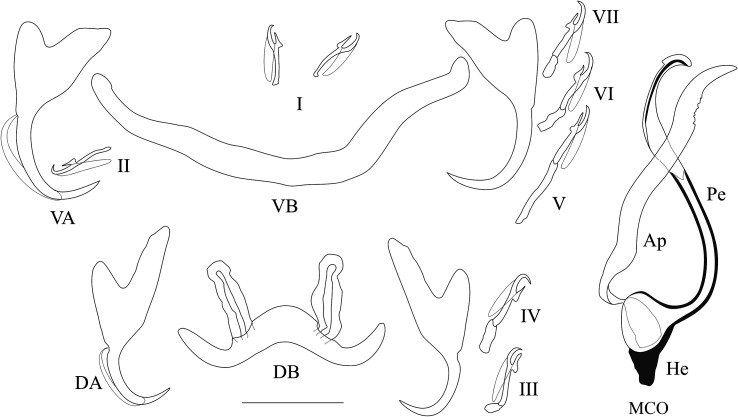
Sclerotised parts (haptor and male copulatory organ) of *Cichlidogyrus furu* n. sp. **I–VII**, hook pairs; **DA**, dorsal anchors; **DB**, dorsal transverse bar; **VA**, ventral anchors; **VB**, ventral transverse bar. **MCO**, male copulatory organ: **AP**, accessory piece; **Pe**, penis; **He**, heel. Scale bar: 20 μm.

*Description* (Table 3, Fig. 8): Two pairs of anchors of similar size and unequal shape (guard, and length difference between shaft and guard, more pronounced in dorsal anchors). Ventral bar V-shaped with thin branches. Dorsal bar thin with two auricles inserted at its dorsal surface. Hooks 7 pairs; I and III to VII short, except for pair V which is of medium size (see [39, 40]). Penis an S-shaped tube, sclerotised from its basal bulb until halfway its total length, opens at its distal third and ends in a groove; accessory piece simple, attached to the rounded basal bulb of the penis by a thin filament; developed heel. No sclerotised vagina.

**Figure 9:**
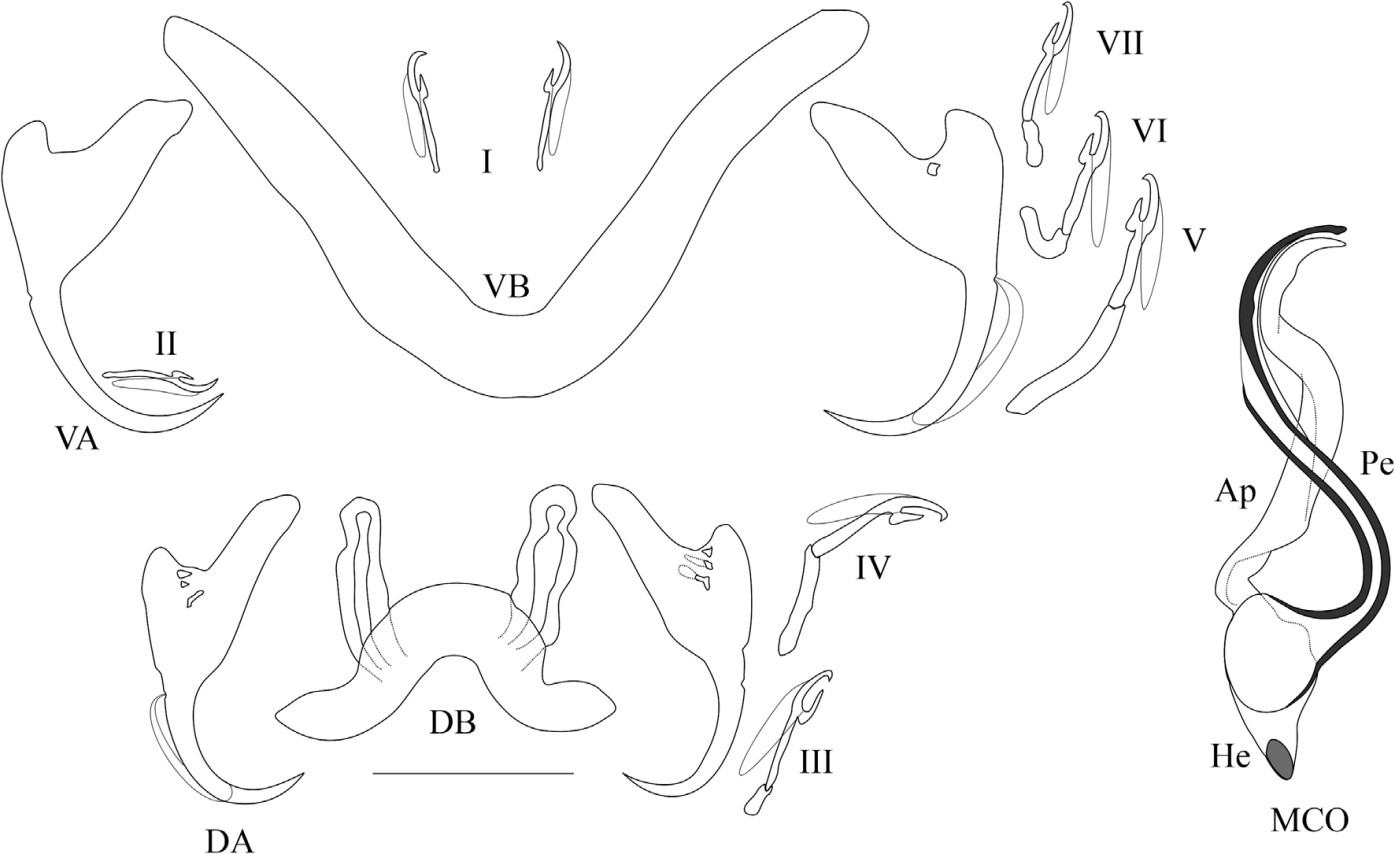
Sclerotised parts (haptor and male copulatory organ) of *Cichlidogyrus* cf. *furu* n. sp. **I–VII**, hook pairs; **DA**, dorsal anchors; **DB**, dorsal transverse bar; **VA**, ventral anchors; **VB**, ventral transverse bar. **MCO**, male copulatory organ: **AP**, accessory piece; **Pe**, penis; **He**, heel. Scale bar: 20 μm.

**Figure 10:**
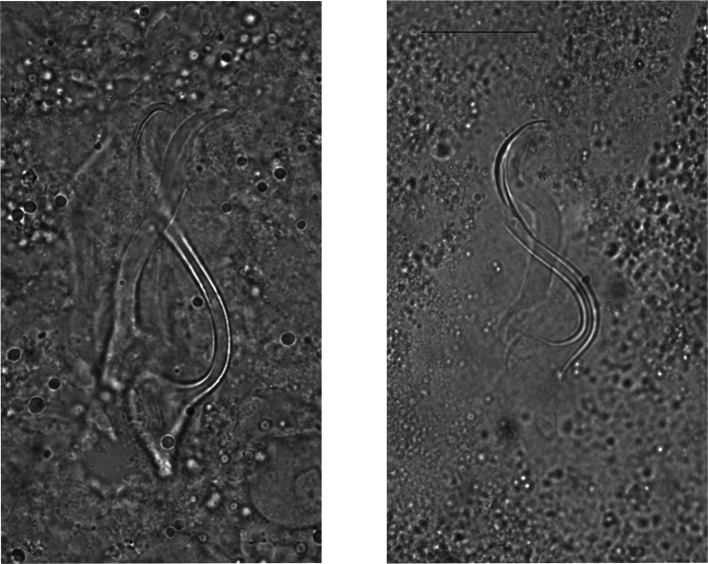
Micrographs of the male copulatory organ of *Cichlidogyrus furu* n. sp. (**left**) and of *C.* cf. *furu* n. sp. (**right**), fixed in Hoyer’s medium. Scale bar: 20 μm.

*Remarks*: *Cichlidogyrus furu* n. sp. shares the short hook pairs III–VII and the non-sclerotised vagina with *C. nyanza* n. sp. and several other congeners infecting haplochromine cichlids (see above). It is unique among species of *Cichlidogyrus* in having a penis ending in a groove. This feature had already been described in other dactylogyridean monogenean species (e.g. *Synodontella speroadotevii* Bouah, N’Douba & Pariselle 2019; [1]). Two specimens (deposited as vouchers under MNHN_HEL1480 and MNHN_HEL1481), taken from one *Pundamilia* sp. ‘pink anal’ from Makobe (stored as 104372 at EAWAG) and one *Pundamilia* sp. ‘nyererei-like’ from Kissenda (stored as 104754 at EAWAG) differ from *C. furu* n. sp. in having the penis entirely sclerotised (Figs. 9 and 10) but were otherwise very similar to *C. furu* n. sp. Since measurements of this morphotype fall into the range of *C. furu* n. sp. and we cannot exclude that the peculiar trait resulted from mounting, we report it as *Cichlidogyrus* cf. *furu* n. sp. and not as a new species.

### *Cichlidogyrus vetusmolendarius* n. sp.

urn:lsid:zoobank.org:act:A526328E-270B-4B95-921C-FB1A3C528586

*Previous records*: *Cichlidogyrus* sp. V sensu Gobbin et al. [15, 16], *C.* sp. “vetusmolendarius” sensu Gobbin et al. [17], see also [14].

*Type host*: *Pseudocrenilabrus multicolor victoriae* Seegers, 1990.

*Type locality*: Off Makobe Island, Lake Victoria.

*Holotype*: MNHN_HEL1483.

*Paratypes*: MNHN_HEL1479, MNHN_HEL1474, RMCA_VERMES_43422.

*Infection site*: Gills.

*Other hosts*: *Mbipia lutea* Seehausen & Bouton 1998; *Pundamilia pundamilia* Seehausen & Bouton, 1998; *Pundamilia nyererei* (Witte-Maas & Witte, 1985); *Pundamilia* sp. ‘nyererei-like’; *Pundamilia* sp. ‘pink anal’; *Pundamilia* sp. ‘pundamilia-like’*.*

*Other localities*: Kissenda Island, Sweya swampy inlet.

*Infection parameters*: Makobe: *Pundamilia nyererei* P = 1.5% (1/65), IF = 1; *P. pundamilia* P = 1.6% (1/62), IF = 1; *Pundamilia* sp. ‘pink anal’ P = 7.1% (1/14), IF = 1; Kissenda: *Pundamilia* sp. ‘nyererei-like’ P = 6.2% (1/16), IF = 2; *Pundamilia* sp. ‘pundamilia-like’ P = 5.5% (1/18), IF = 1; Sweya: “*Astatotilapia” nubila* P = 6.7% (1/15), IF = 2 *Pseudocrenilabrus multicolor victoriae* P = 11.5% (3/26), IF = 1.

*Material studied*: 10 whole-mounted specimens in Hoyer’s solution.

*Symbiotype*: *Pseudocrenilabrus multicolor victoriae* Seegers, 1990 (EAWAG ID 106984, Table S1).

*Symbioparatypes*: “*Astatotilapia” nubila* (Boulenger, 1906) from Sweya (EAWAG ID 109405, Table S1); *Mbipia lutea* Seehausen & Bouton 1998 from Makobe (EAWAG ID 13313, Table S1); *Pseudocrenilabrus multicolor victoriae* Seegers, 1990 (EAWAG ID 105869, Table S1).

*Etymology*: The species epithet refers to the type host, a representative of a relatively old haplochromine lineage compared to most other hosts studied (“vetus”, Latin adjective meaning “old”), and to the shape of the first pair of hooks, similar to the wings of a windmill (“molendarius”, Latin adjective derived from “molendinum”).

**Figure 11:**
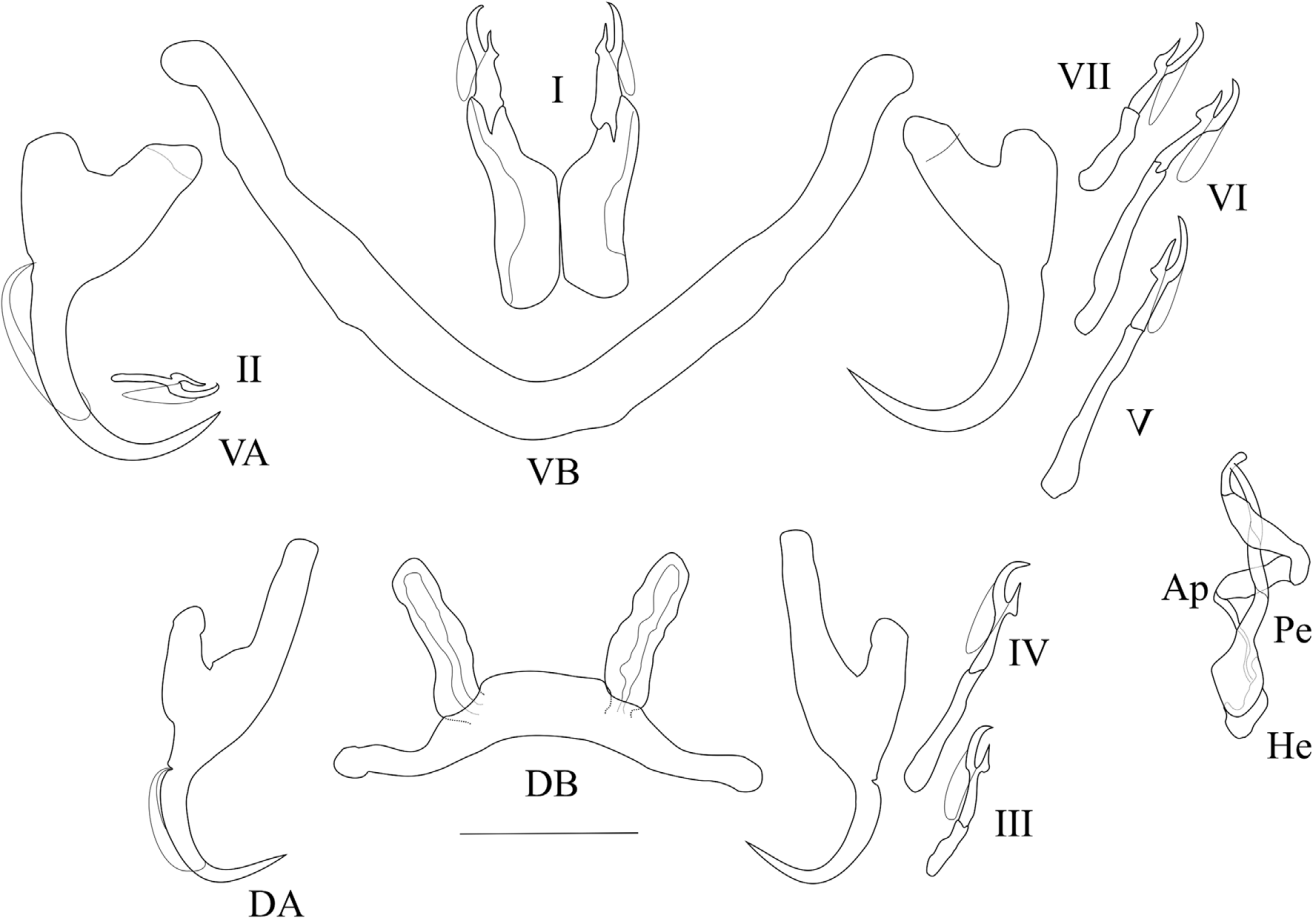
Sclerotised parts (haptor and male copulatory organ) of *Cichlidogyrus vetusmolendarius* n. sp. **I–VII**, hook pairs; **DA**, dorsal anchors; **DB**, dorsal transverse bar; **VA**, ventral anchors; **VB**, ventral transverse bar. **MCO**, male copulatory organ: **AP**, accessory piece; **Pe**, penis; **He**, heel. Scale bar: 20 μm.

*Description* (Table 3, Fig. 11): Two pairs of anchors of unequal shape and size (guard longer in dorsal anchors). Ventral bar long and thin, V-shaped. Dorsal bar thin with two auricles inserted at its dorsal surface. Hooks 7 pairs; I thick and long with a large shaft; III and VII short (22.8 and 23.3 μm), IV to VI longer (27.3, 31.0 and 28.0 μm) (see [39, 40]). Penis short, thin, with a straight direction and spirally coiled on itself (1.5 turns). Accessory piece simple, winding around the penis, and attached to the basal bulb of the penis by a thin and spirally coiled filament (1.5 turns). Poorly developed heel. No sclerotised vagina.

*Remarks*: This new species belongs to the group showing the following characters: hooks pair I very large, no visible vagina. The only previously described species from Lake Victoria haplochromines that falls into this morphological group is *C. dionchus* Paperna & Thurston, 1969, which differs from *C. vetusmolendarius* n. sp. in the shape of its accessory piece (which is curved instead of coiled in *C. vetusmolendarius* n. sp., and broadens terminally and subsequently ends in a hook-like extremity), its clearly elongated heel (poorly developed in *C. vetusmolendarius* n. sp.) and its dorsal anchors with proportionally longer point and blade. This group also comprises: *C. arfii* Pariselle & Euzet, 1995 (on a pelmatolapiine in West Africa), *C. berradae* Pariselle & Euzet, 2003 (on a few coptodonine and a pelmatolapiine species of Central Africa), *C. bulbophallus* Geraerts & Muterezi Bukinga, 2020 (on a haplochromine species in Central Africa), *C. centesimus* Vanhove, Volckaert & Pariselle, 2011 (on a few ectodines in East Africa), *C. digitatus* Dossou, 1982 (on several coptodonine, a pelmatolapiine and a tilapiine species in Central and West Africa), *C. halinus* Paperna, 1969 (on an oreochromine species in West Africa), *C. maeander* Geraerts & Muterezi Bukinga, 2020 (on a haplochromine and a tilapiine species in Central Africa), *C. nuniezi* Pariselle & Euzet, 1998 (on a tilapiine species in West Africa), *C. papernastrema* Price, Peebles & Bamford, 1969 (on a coptodonine, a tilapiine and an oreochromine species in Central and Southern Africa), *C. philander* Douëllou, 1993 (on a haplochromine in Southern Africa), *C. quaestio* Douëllou, 1993 (on a few haplochromine, a tilapiine and a coptodonine species in Southern Africa), and *C. yanni* Pariselle & Euzet, 1995 (on several coptodonine species and a pelmatolapiine species in West Africa). Among these species, only *C. maeander* has a spirally coiled accessory piece winding around the penis like *C. vetusmolendarius* n. sp. Both species share features of the haptoral sclerotised parts, namely: guards at least twice as long as shafts in both anchors, with the guard more pronounced in the dorsal anchors; dorsal anchors on average longer than ventral ones; auricles implanted at the dorsal surface of the dorsal bar; long first hook pair. Also their accessory pieces resemble each other. *Cichlidogyrus vetusmolendarius* n. sp. differs from *C. maeander* in ventral anchor length (29–41 vs 43–44 μm, respectively), and in the shape of the MCO. In *C. vetusmolendarius* n. sp., the MCO consists in a penis that is short, thin, straight and spirally coiled on itself (1.5 turns), and that displays a poorly developed heel; whereas in *C. maeander* it is described as “penis [stylet] short, forming enlarged bulb at base; base attached to pronounced heel; penis [stylet] distally curved, with pointed end” [12].

## Discussion

All the cichlid species sampled are host to *Cichlidogyrus* spp. (Monopisthocotyla), *Lamproglena monodi* Capart, 1944 (Copepoda), *Ergasilus lamellifer* Fryer, 1961 (Copepoda), and glochidia larvae of freshwater mussels (Bivalvia) attached to their gills [15, 22]. Of the hosts, *A. alluaudi* has previously been investigated in taxonomic work for its monogenean gill parasites, next to nine members of the haplochromine radiation and four non-haplochromine cichlid species present in Lake Victoria (overview in [42]). Hence, our survey more than doubles the number of cichlid species from Lake Victoria that have been scrutinised for their gill parasitofauna and their monogenean parasites identified to species level or formally described. This expansion of host coverage, although not including a formal description at the time, already allowed Gobbin, Vanhove et al*.* [15] to propose several patterns with regard to the diversity of *Cichlidogyrus* in Lake Victoria haplochromines. First, the non-radiating haplochromines harbour a monogenean fauna that is distinct from their radiating counterparts. This is not surprising, given that the non-radiating *A. alluaudi* was the sole host known for the only species of *Cichlidogyrus* that is currently hypothesised to be endemic to Lake Victoria, *C. longipenis*. Second, the dactylogyrid monogeneans infecting members of the haplochromine radiation were rarely found on cichlids not belonging to the radiation, in line with the trends observed in the overview of Pariselle, Muterezi Bukinga et al*.* [42].

Recent research on the evolutionary ecology of cichlid-parasite interactions in Lake Victoria proposed that the communities of dactylogyrid flatworms infecting the lake’s haplochromine radiation differ from those parasitising species of two ancient non-radiating haplochromine lineages in the same locations [15]. Cichlid species that are members of the young radiation seemed to share the same set of dactylogyrid flatworm species among each other, but also occasionally with the ancient haplochromine lineages (represented by *A. alluaudi* and *P. multicolor victoriae*). Here, we aimed to underpin these observations with formal taxonomic assessment of the gill monogeneans in question. Of the ten species previously reported in Lake Victoria, we found only two in the present study (*C. bifurcatus* and *C. longipenis*). This can be explained by the difference in locations and/or host lineages surveyed. We surveyed members of Haplochromini from the southern part of Lake Victoria (Tanzania), while previous studies surveyed the northern part (Uganda) and mostly focused on other tribes (such as Oreochromini and Tilapiini) [36–38]. We hypothesise that host lineage, rather than geographic locality, is the more important factor in determining infection variation because the two abovementioned species of *Cichlidogyrus* have been documented in the same host species in the present study and in the previous ones. This study increases the number of nominal monogenean species that are exclusively known from Lake Victoria from one (*C. longipenis*) to five. The fact that only four (or five, when including *C.* cf. *furu* n. sp.) new species are found when characterising the monogenean fauna of 20 host species, from five different locations, is in line with the trends suggested by Pariselle, Muterezi Bukinga et al*.* [42]: the discovery rate and proportion of endemism in *Cichlidogyrus* are lower in the young Lake Victoria compared to the ancient Lake Tanganyika. Indeed, when comparing Lake Victoria’s littoral haplochromines with Tropheini, a lineage of littoral Tanganyika haplochromines, the species richness and uniqueness of their monogenean parasites are starkly lower in the former (see e.g., [59], indicating typically at least one, and up to seven, unique parasite species per host species, with only closely related congeneric hosts, rarely, sharing parasite species). This contrasts with the species richness of the cichlid hosts which is twice higher in Lake Victoria than in Lake Tanganyika [11, 56]. Unlike the haplochromine cichlids in Lake Tanganyika, Lake Victoria haplochromines were now found to also harbour species displaying some long hook pairs (I–VII in *C. pseudodossoui* n. sp., I in *C. vetusmolendarius* n. sp.) and a more complex accessory piece of the MCO (*C. pseudodossoui* n. sp. and *C. vetusmolendarius* n. sp.). Furthermore, it is the first time that a penis ending in an open groove was observed in *Cichlidogyrus*, in *C. furu* n. sp., adding to the known diversity in the morphology of monogeneans infecting African Great Lake haplochromines. Also noteworthy about *Cichlidogyrus furu* n. sp. is that, while the relative lengths of hook pairs III to VII were previously often considered together [39, 61], in this species, pair V is clearly longer than the others. Size differences within hook pairs III to VII have also been reported from recently discovered congeners in the Congo river basin [12, 21]. In agreement with Rahmouni, Vanhove et al*.* [45] and Geraerts, Muterezi Bukinga et al*.* [12], this further illustrates that the different haptor configurations proposed for the classification of species of *Cichlidogyrus* into four morphotype groups, mostly based on West African representatives (see [43, 61]) cannot accommodate all the recently described species from Central, East and Southern Africa. Phylogenetic analyses with increased species coverage indicated that the length of the haptoral hooks is systematically informative [7]. The genetic data required for this could also help in clarifying whether some morphological features could be key to understand the shared history of these species. This may be the case for the tube-shaped penis and simple accessory piece of *C. bifurcatus*, *C. longipenis*, and *C. nyanza* n. sp., that are shared with many congeners infecting haplochromines (see [13, 34, 57]), and for the long hooks V shared by *C. furu* n. sp., *C. calycinus* Kusters, Jorissen, Pariselle & Vanhove, 2018, and *C. omari* Jorissen, Pariselle & Vanhove, 2018 [21]. Pending confirmation of this phylogenetic signal, we note the morphological similarity of these species with typical parasites of haplochromines (reported from representatives of e.g. *Haplochromis* Hilgendorf, 1888, *Orthochromis* Greenwood, 1954, *Pharyngochromis* Greenwood, 1979, *Pseudocrenilabrus* Fowler, 1934, *Sargochromis* Regan, 1920, *Serranochromis* Regan, 1920 and Tropheini), namely a simple male copulatory organ and similar size and shape of ventral and dorsal anchors. On the other hand, *C. pseudodossoui* n. sp. resembles species described from mainly West African cichlid hosts belonging to Heterotilapiini, Pelmatolapiini, Gobiocichlini, Coptodonini, and Oreochromini, and hence probably belongs to an entirely different lineage of *Cichlidogyrus*. This suggests that members of *Cichlidogyrus* colonised Lake Victoria haplochromines or their ancestors at least twice. Lake Victoria was colonised by at least four lineages belonging to two tribes (Haplochromini and Oreochromini) [30]. The taxonomic coverage of the current phylogenetic reconstruction for *Cichlidogyrus* across Africa does not suffice to test whether this provides a likely pathway for the origins of the Lake Victoria cichlid monogeneans. However, in the context of the phylogeny of *Cichlidogyrus*, we can already observe that the haptors of the Lake Victoria parasites have auricles attached to the dorsal side of the dorsal bar. This is considered a derived state, in contrast to the auricles being a continuation of the anterior side of the dorsal bar, as in the earlier diverged parasites of the only very distantly related tylochromine cichlids [33]. While the dominance of *C. bifurcatus* on *P. multicolor victoriae*, and of *C. longipenis* on *A. alluaudi*, in contrast to the communities of *Cichlidogyrus* of the other investigated hosts, clearly demonstrate community-level differences between member species of the haplochromine radiation and other Lake Victoria haplochromines, none of these monogeneans seems specialised to a single host species. We concur with Pariselle, Muterezi Bukinga et al*.* [42] that this low host-specificity may be a consequence of the young age of the lake and its fish assemblage. It would therefore be interesting to expand sampling to adjacent lakes and river systems, to test the hypothesis that their haplochromine cichlids harbour the same or closely related species of *Cichlidogyrus* [30, 64].

## Supplementary material

The supplementary material for this article can be found at https://www.parasite-journal.org/10.1051/parasite/2024039/olm. *Table S1:* Host species, identification number (according to FishEc collection at Eawag, Switzerland), location (island at southern Lake Victoria), date of capture and death, capture water depth (m) of cichlid specimens that harboured the species of *Cichlidogyrus* (re)described in this study.
