# Supplementary material for: Four new species of Cichlidogyrus (Platyhelminthes, Monopisthocotyla, Dactylogyridae) from Lake Victoria haplochromine cichlid fishes, with the redescription of C. bifurcatus and C. longipenis
Source: Parasite. 2024 Aug 7;31:46. doi: 10.1051/parasite/2024039 (PMC11305117; doi:10.1051/parasite/2024039)
Supplement: Supplementary file 1 — Table S1: Host species, identification number (according to FishEc collection at Eawag, Switzerland), location (island at southern Lake Victoria), date of capture and death, capture water depth (m) of cichlid specimens that harboured the species of Cichlidogyrus (re)described in this study. [file parasite-31-46-s1.pdf]

Four new species of *Cichlidogyrus* (Platyhelminthes, Monopisthocotyla, Dactylogyridae) from Lake Victoria haplochromine cichlid fishes, with the redescription of *C. bifurcatus* and *C. longipenis*

Tiziana P Gobbin, Maarten PM Vanhove, Ole Seehausen, Martine E Maan, Antoine Pariselle

SUPPLEMENTARY MATERIAL

Table S1

Host species, identification number (according to FishEc collection at Eawag, Switzerland), location (island at southern Lake Victoria), date of capture and death, capture water depth (m) of cichlid specimens that harboured the species of *Cichlidogyrus* (re)described in this study.

ID	Species	Island	Date of death	Depth (m)
103148	*Astatoreochromis alluaudi*	Makobe	13.10.2014	9.00
103216	*Astatoreochromis alluaudi*	Makobe	13.10.2014	18.50
103385	*Astatoreochromis alluaudi*	Makobe	20.10.2014	16.00
103567	*Astatoreochromis alluaudi*	Makobe	20.10.2014	0.75
103571	*Astatoreochromis alluaudi*	Makobe	20.10.2014	0.75
106791	*Astatoreochromis alluaudi*	Makobe	29.09.2014	0.75
106796	*Astatoreochromis alluaudi*	Makobe	29.09.2014	12.50
106812	*Astatoreochromis alluaudi*	Makobe	29.09.2014	12.50
109119	*Astatoreochromis alluaudi*	Makobe	25.10.2014	7.50
103674	*Astatoreochromis alluaudi*	Sweya	23.10.2014	0.50
103677	*Astatoreochromis alluaudi*	Sweya	23.10.2014	0.50
103881	*Astatoreochromis alluaudi*	Sweya	22.10.2014	0.50
105845	*Astatoreochromis alluaudi*	Sweya	28.09.2014	0.50
103309	*Paralabidochromis chilotes*	Makobe	17.10.2014	13.00
105235	*Paralabidochromis chilotes*	Makobe	29.09.2014	6.50
105334	*Paralabidochromis chilotes*	Makobe	10.06.2014	7.00
106795	*Paralabidochromis chilotes*	Makobe	29.09.2014	12.50
105128	*“Haplochromis” cyaneus*	Makobe	13.10.2014	2.50
105317	*“Haplochromis” cyaneus*	Makobe	10.06.2014	2.50
105319	*“Haplochromis” cyaneus*	Makobe	10.06.2014	3.50
105323	*“Haplochromis” cyaneus*	Makobe	10.06.2014	2.50
105331	*“Haplochromis” cyaneus*	Makobe	10.06.2014	2.50
109405	*Astatotilapia nubila*	Sweya	26.10.2014	0.50
10785	*Ptyochromis xenognathus*	Kissenda	01.06.2010	1.50
12048	*Ptyochromis xenognathus*	Kissenda	01.07.2010	3.00
12073	*Ptyochromis xenognathus*	Kissenda	01.07.2010	na
12306	*Ptyochromis xenognathus*	Kissenda	07.07.2010	1.60
14357	*Labrochromis* sp. ‘stone’	Makobe	06.08.2010	16.00
14557	*Labrochromis* sp. ‘stone’	Makobe	13.08.2010	15.00
13313	*Mbipia lutea*	Makobe	23.07.2010	0.75
13801	*Mbipia lutea*	Makobe	25.07.2010	na
13839	*Mbipia lutea*	Makobe	27.07.2010	1.50
10242	*Mbipia mbipi*	Makobe	22.05.2010	1.50
11348	*Mbipia mbipi*	Makobe	10.06.2010	0.75
12951	*Mbipia mbipi*	Makobe	19.07.2010	1.00
105212	*Mbipia mbipi*	Makobe	24.09.2014	2.00
105213	*Mbipia mbipi*	Makobe	24.09.2014	2.00
105217	*Mbipia mbipi*	Makobe	24.09.2014	2.00
105223	*Mbipia mbipi*	Makobe	25.09.2014	2.50
105338	*Mbipia mbipi*	Makobe	10.06.2014	1.00
106162	*Mbipia mbipi*	Makobe	24.09.2014	0.75
106278	*Mbipia mbipi*	Makobe	24.09.2014	1.25
106279	*Mbipia mbipi*	Makobe	24.09.2014	1.25

ID	Species	Island	Date of death	Depth (m)
105114	*Neochromis gigas*	Makobe	13.10.2014	1.00
105305	*Neochromis gigas*	Makobe	10.06.2014	1.00
106160	*Neochromis gigas*	Makobe	24.09.2014	2.75
103154	*Neochromis omnicaeruleus*	Makobe	13.10.2014	9.50
103562	*Neochromis omnicaeruleus*	Makobe	20.10.2014	4.25
103574	*Neochromis omnicaeruleus*	Makobe	20.10.2014	4.25
103577	*Neochromis omnicaeruleus*	Makobe	20.10.2014	4.25
104392	*Neochromis omnicaeruleus*	Makobe	06.10.2014	6.00
104562	*Neochromis omnicaeruleus*	Makobe	06.10.2014	3.00
104569	*Neochromis omnicaeruleus*	Makobe	06.10.2014	3.00
105124	*Neochromis omnicaeruleus*	Makobe	13.10.2014	2.50
105655	*Neochromis omnicaeruleus*	Makobe	19.09.2014	5.00
109059	*Neochromis omnicaeruleus*	Makobe	25.10.2014	7.00
109211	*Neochromis omnicaeruleus*	Makobe	27.10.2014	7.00
109212	*Neochromis omnicaeruleus*	Makobe	27.10.2014	7.00
109214	*Neochromis omnicaeruleus*	Makobe	27.10.2014	7.00
104539	*Neochromis rufocaudalis*	Makobe	06.10.2014	0.75
105119	*Neochromis rufocaudalis*	Makobe	13.10.2014	2.50
105120	*Neochromis rufocaudalis*	Makobe	13.10.2014	2.50
106280	*Neochromis rufocaudalis*	Makobe	24.09.2014	1.25
103155	*Neochromis* sp. 'unicuspid scraper'	Makobe	13.10.2014	9.50
103221	*Neochromis* sp. 'unicuspid scraper'	Makobe	13.10.2014	18.50
103223	*Neochromis* sp. 'unicuspid scraper'	Makobe	13.10.2014	18.50
103319	*Neochromis* sp. 'unicuspid scraper'	Makobe	17.10.2014	16.00
103335	*Neochromis* sp. 'unicuspid scraper'	Makobe	17.10.2014	16.00
103583	*Neochromis* sp. 'unicuspid scraper'	Makobe	20.10.2014	11.00
104366	*Neochromis* sp. 'unicuspid scraper'	Makobe	06.10.2014	19.00
104368	*Neochromis* sp. 'unicuspid scraper'	Makobe	06.10.2014	19.00
104370	*Neochromis* sp. 'unicuspid scraper'	Makobe	06.10.2014	19.00
104371	*Neochromis* sp. 'unicuspid scraper'	Makobe	06.10.2014	19.00
104373	*Neochromis* sp. 'unicuspid scraper'	Makobe	06.10.2014	19.00
104405	*Neochromis* sp. 'unicuspid scraper'	Makobe	06.10.2014	8.00
104408	*Neochromis* sp. 'unicuspid scraper'	Makobe	06.10.2014	8.00
104553	*Neochromis* sp. 'unicuspid scraper'	Makobe	06.10.2014	17.00
104554	*Neochromis* sp. 'unicuspid scraper'	Makobe	06.10.2014	17.00
104557	*Neochromis* sp. 'unicuspid scraper'	Makobe	06.10.2014	17.00
109124	*Neochromis* sp. 'unicuspid scraper'	Makobe	25.10.2014	7.50
109135	*Neochromis* sp. 'unicuspid scraper'	Makobe	25.10.2014	9.50
109138	*Neochromis* sp. 'unicuspid scraper'	Makobe	25.10.2014	9.50
109140	*Neochromis* sp. 'unicuspid scraper'	Makobe	25.10.2014	9.50
105867	*Pseudocrenilabrus multicolor victoriae*	Sweya	12.10.2014	0.50
105869	*Pseudocrenilabrus multicolor victoriae*	Sweya	12.10.2014	0.50
106984	*Pseudocrenilabrus multicolor victoriae*	Sweya	05.10.2014	0.50
106986	*Pseudocrenilabrus multicolor victoriae*	Sweya	05.10.2014	0.50
109436	*Pseudocrenilabrus multicolor victoriae*	Sweya	28.10.2014	0.50
109437	*Pseudocrenilabrus multicolor victoriae*	Sweya	28.10.2014	0.50
13057	*Ptyochromis* sp. 'striped rock sheller'	Makobe	19.07.2010	7.50
10964	*Pundamilia nyererei*	Makobe	31.05.2010	8.75
11099	*Pundamilia nyererei*	Makobe	04.06.2010	7.25
103266	*Pundamilia nyererei*	Makobe	13.10.2014	18.50
103275	*Pundamilia nyererei*	Makobe	13.10.2014	18.50
103279	*Pundamilia nyererei*	Makobe	13.10.2014	18.50
103306	*Pundamilia nyererei*	Makobe	17.10.2014	13.00
103312	*Pundamilia nyererei*	Makobe	17.10.2014	16.00
103397	*Pundamilia nyererei*	Makobe	20.10.2014	16.00
103588	*Pundamilia nyererei*	Makobe	20.10.2014	11.00
104317	*Pundamilia nyererei*	Makobe	06.10.2014	2.50
104428	*Pundamilia nyererei*	Makobe	06.10.2014	12.75
104433	*Pundamilia nyererei*	Makobe	06.10.2014	12.75
104474	*Pundamilia nyererei*	Makobe	06.10.2014	8.00
105263	*Pundamilia nyererei*	Makobe	29.09.2014	10.00

ID	Species	Island	Date of death	Depth (m)
105660	*Pundamilia nyererei*	Makobe	19.09.2014	6.00
106128	*Pundamilia nyererei*	Makobe	24.09.2014	9.00
106255	*Pundamilia nyererei*	Makobe	24.09.2014	9.50
106830	*Pundamilia nyererei*	Makobe	29.09.2014	12.50
106884	*Pundamilia nyererei*	Makobe	29.09.2014	14.50
109143	*Pundamilia nyererei*	Makobe	25.10.2014	9.50
109291	*Pundamilia nyererei*	Makobe	29.10.2014	7.00
10947	*Pundamilia pundamilia*	Makobe	04.06.2010	1.63
103165	*Pundamilia pundamilia*	Makobe	13.10.2014	11.25
104395	*Pundamilia pundamilia*	Makobe	06.10.2014	0.75
104397	*Pundamilia pundamilia*	Makobe	06.10.2014	0.75
104400	*Pundamilia pundamilia*	Makobe	06.10.2014	0.75
105605	*Pundamilia pundamilia*	Makobe	19.09.2014	0.75
105679	*Pundamilia pundamilia*	Makobe	19.10.2014	0.75
106163	*Pundamilia pundamilia*	Makobe	24.09.2014	0.75
106272	*Pundamilia pundamilia*	Makobe	24.09.2014	0.75
106274	*Pundamilia pundamilia*	Makobe	24.09.2014	0.50
106284	*Pundamilia pundamilia*	Makobe	24.09.2014	0.75
106290	*Pundamilia pundamilia*	Makobe	29.10.2014	6.00
106719	*Pundamilia pundamilia*	Makobe	29.09.2014	6.00
106888	*Pundamilia pundamilia*	Makobe	29.09.2014	0.75
106889	*Pundamilia pundamilia*	Makobe	29.09.2014	0.75
109145	*Pundamilia pundamilia*	Makobe	25.10.2014	1.00
109147	*Pundamilia pundamilia*	Makobe	25.10.2014	1.00
109148	*Pundamilia pundamilia*	Makobe	25.10.2014	1.00
109156	*Pundamilia pundamilia*	Makobe	25.10.2014	0.75
109332	*Pundamilia pundamilia*	Makobe	29.10.2014	0.75
109337	*Pundamilia pundamilia*	Makobe	29.10.2014	0.75
103668	*Pundamilia* sp. ‘Luanso’ blue	Luanso	23.10.2014	1.00
103669	*Pundamilia* sp. ‘Luanso’ blue	Luanso	23.10.2014	1.00
103671	*Pundamilia* sp. ‘Luanso’ blue	Luanso	23.10.2014	1.00
105804	*Pundamilia* sp. ‘Luanso’ blue	Luanso	22.09.2014	0.75
105814	*Pundamilia* sp. ‘Luanso’ blue	Luanso	22.09.2014	3.50
105847	*Pundamilia* sp. ‘Luanso’ blue	Luanso	10.11.2014	4.00
105855	*Pundamilia* sp. ‘Luanso’ blue	Luanso	10.11.2014	1.50
105801	*Pundamilia* sp. ‘Luanso’ red	Luanso	22.09.2014	0.75
105813	*Pundamilia* sp. ‘Luanso’ red	Luanso	22.09.2014	3.75
105817	*Pundamilia* sp. ‘Luanso’ red	Luanso	26.09.2014	1.50
105819	*Pundamilia* sp. ‘Luanso’ red	Luanso	26.09.2014	1.50
105828	*Pundamilia* sp. ‘Luanso’ red	Luanso	26.09.2014	1.50
105851	*Pundamilia* sp. ‘Luanso’ red	Luanso	10.11.2014	4.00
104734	*Pundamilia* sp. ‘nyererei-like’	Kissenda	14.10.2014	1.75
104738	*Pundamilia* sp. ‘nyererei-like’	Kissenda	14.10.2014	1.75
104739	*Pundamilia* sp. ‘nyererei-like’	Kissenda	14.10.2014	1.75
104753	*Pundamilia* sp. ‘nyererei-like’	Kissenda	14.10.2014	5.50
104754	*Pundamilia* sp. ‘nyererei-like’	Kissenda	14.10.2014	5.50
104765	*Pundamilia* sp. ‘nyererei-like’	Kissenda	14.10.2014	7.50
104768	*Pundamilia* sp. ‘nyererei-like’	Kissenda	14.10.2014	7.50
104769	*Pundamilia* sp. ‘nyererei-like’	Kissenda	14.10.2014	3.00
105341	*Pundamilia* sp. ‘nyererei-like’	Kissenda	10.07.2014	3.00
105348	*Pundamilia* sp. ‘nyererei-like’	Kissenda	10.07.2014	3.00
105349	*Pundamilia* sp. ‘nyererei-like’	Kissenda	10.07.2014	3.00
105351	*Pundamilia* sp. ‘nyererei-like’	Kissenda	10.07.2014	3.00
105712	*Pundamilia* sp. ‘nyererei-like’	Kissenda	20.09.2014	0.75
106059	*Pundamilia* sp. ‘nyererei-like’	Kissenda	30.09.2014	4.00
103340	*Pundamilia* sp. ‘pink anal’	Makobe	17.10.2014	16.00
103545	*Pundamilia* sp. ‘pink anal’	Makobe	20.10.2014	16.00
104372	*Pundamilia* sp. ‘pink anal’	Makobe	06.10.2014	19.00
105239	*Pundamilia* sp. ‘pink anal’	Makobe	29.09.2014	7.50
106806	*Pundamilia* sp. ‘pink anal’	Makobe	29.09.2014	12.50
109178	*Pundamilia* sp. ‘pink anal’	Makobe	27.10.2014	5.50

ID	Species	Island	Date of death	Depth (m)
109221	*Pundamilia* sp. ‘pink anal’	Makobe	27.10.2014	14.00
104710	*Pundamilia* sp. ‘pundamilia-like’	Kissenda	14.10.2014	5.50
104711	*Pundamilia* sp. ‘pundamilia-like’	Kissenda	14.10.2014	7.50
104722	*Pundamilia* sp. ‘pundamilia-like’	Kissenda	14.10.2014	3.00
104746	*Pundamilia* sp. ‘pundamilia-like’	Kissenda	14.10.2014	3.00
105346	*Pundamilia* sp. ‘pundamilia-like’	Kissenda	10.07.2014	7.50
105353	*Pundamilia* sp. ‘pundamilia-like’	Kissenda	10.07.2014	2.00
105355	*Pundamilia* sp. ‘pundamilia-like’	Kissenda	10.07.2014	1.00
105356	*Pundamilia* sp. ‘pundamilia-like’	Kissenda	10.07.2014	1.00
105359	*Pundamilia* sp. ‘pundamilia-like’	Kissenda	10.07.2014	1.00
106928	*Pundamilia* sp. ‘pundamilia-like’	Kissenda	07.10.2014	7.50
106932	*Pundamilia* sp. ‘pundamilia-like’	Kissenda	07.10.2014	7.50
106981	*Pundamilia* sp. ‘pundamilia-like’	Kissenda	07.10.2014	2.00
103821	*Pundamilia* sp. ‘pundamilia-like’	Python	10.10.2014	0.75
